# Identifying and correcting repeat-calling errors in nanopore sequencing of telomeres

**DOI:** 10.1186/s13059-022-02751-6

**Published:** 2022-08-26

**Authors:** Kar-Tong Tan, Michael K. Slevin, Matthew Meyerson, Heng Li

**Affiliations:** 1 Department of Medical Oncology, Dana-Farber Cancer Institute, Boston, MA USA; 2 Cancer Program, Broad Institute of MIT and Harvard, Cambridge, MA USA; 3 Department of Genetics, Harvard Medical School, Boston, MA USA; 4 Center for Cancer Genomics, Dana-Farber Cancer Institute, Boston, MA USA; 5 Department of Data Sciences, Dana-Farber Cancer Institute, Boston, MA USA; 6 Department of Biomedical Informatics, Harvard Medical School, Boston, MA USA

**Keywords:** Nanopore-sequencing, Long-reads, Telomere, Basecalling

## Abstract

Nanopore long-read sequencing is an emerging approach for studying genomes, including long repetitive elements like telomeres. Here, we report extensive basecalling induced errors at telomere repeats across nanopore datasets, sequencing platforms, basecallers, and basecalling models. We find that telomeres in many organisms are frequently miscalled. We demonstrate that tuning of nanopore basecalling models leads to improved recovery and analysis of telomeric regions, with minimal negative impact on other genomic regions. We highlight the importance of verifying nanopore basecalls in long, repetitive, and poorly defined regions, and showcase how artefacts can be resolved by improvements in nanopore basecalling models.

**Supplementary Information:**

The online version contains supplementary material available at 10.1186/s13059-022-02751-6.

## Background

Telomeres are protective caps found on chromosomal ends and are known to play critical roles in a wide range of biological processes and human diseases [1, 2]. These highly repetitive structures enable cells to deal with the “end-replication problem” through the action of telomerase which adds telomeric repeats to the ends of chromosomes. In cancer, the reactivation of telomerase to drive telomere elongation is estimated to occur in as many as 90% of human cancers and has been shown experimentally to be critical for malignant transformation [3–8]. As one ages, telomeres are also known to progressively shorten and are thus thought to also play a central role in the process of aging [9–11]. In many organisms, telomeres are characterized by (TTAGGG)_n_ repeats that vary in length of between 2 and 20 kb long, which are not readily resolved by short-read sequencing approaches. Given the importance of telomeres in a wide range of biological processes and the technical challenges associated with their analysis using short-read sequencing, there is significant interest in applying emerging techniques like long-read sequencing to study these repetitive structures.

Long-read sequencing has emerged as a powerful technology for the study of long repetitive elements in the genome. Two main platforms, Single Molecule Real Time (SMRT) sequencing and nanopore sequencing, have been developed to generate sequence reads of over 10 kilobases from DNA molecules [12, 13]. In SMRT Sequencing, the incorporation of DNA nucleotides is captured real-time via one of four different fluorescent dyes attached to each of the four DNA bases, thereby allowing the corresponding DNA sequence to be inferred. Sequencing of the same DNA molecule multiple times in a circular manner further allows a highly accurate consensus sequence of the DNA molecule to be generated in a process termed Pacific Biosciences (PacBio) High-Fidelity (HiFi) sequencing [12]. During nanopore sequencing, the ionic current, which varies according to the DNA sequence, is measured while a single-stranded DNA molecule passes through a nanopore channel. The electrical current measurement is then converted into the corresponding DNA sequence using a deep neural network trained on a collection of ionic current profiles of known DNA sequences [13]. Notably, both platforms enable long DNA molecules of more than 10 kilobase pairs to be routinely sequenced and are thus highly suited for the study of long repetitive elements like telomeres.

## Results and discussion

**Fig. 1:**
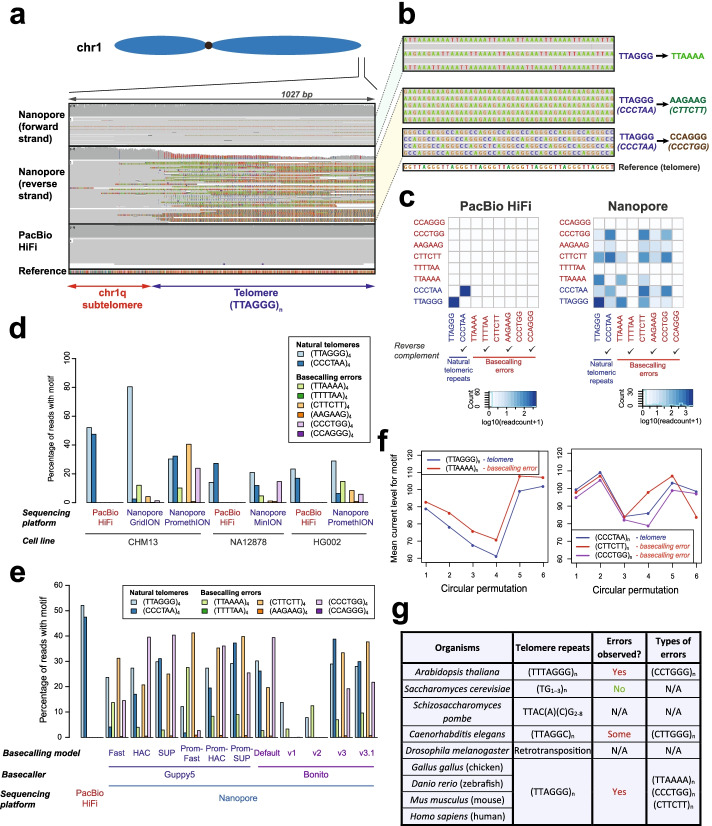
Strand-specific nanopore basecalling errors are pervasive at telomeres. **a**, **b** IGV screenshot illustrating the three types of basecalling errors found on the forward and reverse strands of telomeres for nanopore sequencing. (TTAGGG)_n_ on the forward strand of nanopore sequencing data was basecalled as (TTAAAA)_n_ while (CCCTAA)_n_ on the reverse strand was basecalled as (CTTCTT)_n_ and (CCCTGG)_n_. PacBio HiFi data generated from the same cell line (CHM13) is depicted as a control. Reference genome indicated in the plot corresponds to the chm13 draft genome assembly (v1.0). **c** Co-occurrence heatmap illustrating the frequency of co-occurrence of repeats corresponding to natural telomeres, or to basecalling errors in PacBio HiFi and nanopore long-reads found at chromosomal ends (within 10kb of annotated end of the reference genome). Diagonal of co-occurrence matrix represents counts of long-reads with only a single type of repeats observed. **d** Basecalling errors at telomeres are observed across different nanopore datasets and sequencing platforms. **e** Basecalling errors at telomeres are observed for different nanopore basecallers and basecalling models. Guppy5 and the Bonito basecallers, and different bascalling models for each basecaller, were used to basecall telomeric reads in the CHM13 PromethION dataset (reads that mapped to flanking 10kb regions of the CHM13 reference genome). **f** Basecalling errors share similar nanopore current profiles as telomeric repeats. Current profiles for telomeric and basecalling error repeats were plotted based on known mean current profiles for each k-mer (“Methods”). **g** Summary of organisms assessed and the types of repeat errors observed. Note that *S. pombe* and *D. melanogaster* could not be readily assessed for the presence of error repeats by visualization in IGV as these sequences are more complex

In our analysis of telomeric regions with nanopore long-read sequencing in the recently sequenced and assembled CHM13 sample [14, 15], we surprisingly observed that telomeric regions were frequently miscalled as other types of repeats in a strand-specific manner. Specifically, although human telomeres are typically represented by (TTAGGG)_n_ repeats (Additional file 1: Fig. S1a), these regions were frequently recorded as (TTAAAA)_n_ repeats (Fig. 1a, b, Additional file 1: Fig. S1 and S2a). At the same time, when examining the reverse complementary strand of the telomeres which are represented as (CCCTAA)_n_ repeats, we instead observed frequent substitution of these regions by (CTTCTT)_n_ and (CCCTGG)_n_ repeats (Fig. 1a, b, Additional file 1: Fig. S1 and S2b,c). Notably, these artefacts were not observed on the CHM13 reference genome [14, 15], or PacBio HiFi reads from the same site (Fig. 1a, b), suggesting that these observed repeats are artefacts of nanopore sequencing or the base-calling process, rather than real biological variations of telomeres. The examination of each telomeric long read also indicates that these error repeats frequently co-occur with telomeric repeats at the ends of each read (Fig. 1c, Additional file 1: Fig. S3), and are observed on all chromosomal arms of CHM13 (Additional file 1: Fig. S1b,c, Additional file 1: Fig. S4). Together, our results suggest that telomeric regions are frequently misrepresented as other types of repeats in a strand-specific manner during nanopore sequencing.

As human sub-telomeres are known to have a high degree of similarity to each other [16] which may lead to mis-mapping of reads between different chromosomal arms, we explored the level of read mis-mapping between different arms to assess if this might affect our analysis. We simulated long-reads (mean = 10kb) from the terminal 10 kb, 100 kb, and 1000 kb region of the CHM13 reference genome (Methods) and remapped them to the CHM13 assembly to measure the rate of misalignment. Remarkably, under a mapping quality threshold of ≥1, the mapping error rate was only ~0.03–0.3% for reads ranging in base accuracy between 95 and 99.9% at each of these regions (Additional file 1: Fig. S5). Even when a less stringent mapping quality cutoff value of 0 was applied, a relatively low mapping error rate of 0.3–1.2% was observed (Additional file 1: Fig. S5). As such, our results from reads simulation suggest that there is minimal level of read mis-mapping between different chromosomal arms in the CHM13 sample. We next assessed the sequencing coverage of each chromosomal arm in the CHM13 sample to establish if there may be biases in read coverage caused by read mis-mapping. We did not see strong biases in the coverage of nanopore reads on each chromosomal arm in the CHM13 sample (Additional file 1: Fig. S6), in line with the low mapping error rate in our simulation study. To evaluate if these errors are broadly observed in other studies or are specific to the CHM13 dataset from the Telomere-to-Telomere consortium, we examined the previously published NA12878 and HG002 nanopore genome sequencing datasets [12, 13, 17, 18]. We observed the same basecalling errors, TTAGGG➔TTAAAA, CCCTAA➔CTTCTT, and CCCTAA➔CCCTGG at telomeres in these datasets (Fig. 1d, Additional file 1: Fig. S7a). Remarkably, between 40 and 60% of reads at telomeric regions in these three datasets display at least one of these types of basecalling repeat artefacts for the nanopore sequencing platform (Additional file 1: Fig. S7b), while these errors were not observed in the PacBio HiFi datasets for the same samples (Additional file 1: Fig. S7b). We also partitioned these datasets based on the sequencing platforms used to generate them and noted that basecalling error repeats are observed across all three nanopore sequencing platforms (MinION, GridION, PromethION) (Fig. 1d, Additional file 1: Fig. S7a). These error repeats are a pervasive problem across nanopore sequencing datasets and sequencing platforms.

We then questioned if these error repeats are unique to specific nanopore basecallers or basecalling models. We extracted reads from chromosomal ends, and re-basecalled ionic current data of these reads using different basecallers and basecalling models. Using the production-ready basecaller Guppy5 (Oxford Nanopore Technologies), and the developmental-phase basecaller Bonito (Oxford Nanopore Technologies), we noticed that these basecalling error repeats can be readily observed across both basecallers (Fig. 1e, Additional file 1: Fig. S8 and S9). Further, these error repeats were also observed when different basecalling models were applied (Fig. 1e). Significantly, we also observed that the “fast” basecalling mode in Guppy led to almost complete loss of the (CCCTAA)_n_ strand (Fig. 1e, Additional file 1: Fig. S8a), while the “HAC” basecalling model enabled both strands to be recovered, highlighting that the basecalling model applied can affect the strand-specific recovery of telomeric reads. Together, these results suggest that error repeats are observable across nanopore basecallers and basecalling models.

We asked if there might be a difference in current profiles between the error-prone and less error-prone reads. To distinguish the error-prone reads from the less error-prone reads, we calculated the number of telomeric repeats ((TTAGGG)_3_ and (CCCTAA)_3_), and artefact repeats ((TTAAAA)_3_, (CCCTGG)_3_, and (CTTCTT)_3_) on each long-read (Additional file 1: Fig. S10a-b). The proportion of repeat-calling errors on each read can then be established by dividing the number of artefact repeats by the total number of telomeric and artefact repeats (Additional file 1: Fig. S10c-d). While the majority of long-reads (69.5%) on the “CCCTAA” strand had an error proportion of >90%, only 5.2% of long-reads on the “TTAGGG” strand had an error proportion of <10%, suggesting that the repeat calling errors occur more frequently on the “CCCTAA” strand than on the “TTAGGG” strand. We then examined the current profiles of the more error-prone reads (i.e., reads with a higher proportion of repeat calling errors) and the less error-prone reads. We were not able to observe an obvious visual difference in current profiles between the reads with a higher proportion (>0.9) of repeat calling errors (Additional file 1: Fig. S11a-c) versus the reads with a lower proportion (<0.4) of repeat calling errors (Additional file 1: Fig. S11d-f).

To determine the cause for these repeat-calling errors, we examined the ionic current profiles of true telomeric repeats and artifactual error repeats. We extracted known mean current values of each 6-mer and its six circular permutations (e.g., TTAGGG, TAGGGT, and AGGTT) and generated their ionic current profiles (Methods). Remarkably, we observed a high degree of similarity between current profiles between telomeric repeats and these basecalling errors (Fig. 1f). Specifically, we observed that (TTAGGG)_n_ telomeric repeats had a high degree of similarity with the (TTAAAA)_n_ error repeats generated by the Bonito base-caller (Pearson correlation = 0.9928, Euclidean distance=4.9934) (Additional file 1: Fig. S12a-c). Similarly, (CCCTAA)_n_ current profile also showed high similarity with (CCCTGG)_n_ repeats (Pearson correlation = 0.9783, Euclidean distance = 4.687), and reasonably good similarity with (CTTCTT)_n_ repeats (Pearson correlation = 0.6411, Euclidean distance = 19.384) (Additional file 1: Fig. S12a-c). Together, these results suggest that similarities in current profiles between repeat sequences are possible causes for repeat-calling errors at telomeric repeats.

We then examined if repeat-calling errors may extend to other repetitive sequences beyond telomeric sequences. To address this, we search for other repeat pairs with similar current profiles that may be susceptible to these repeat-calling errors. We simulated and performed pairwise comparison of current profiles for all 6-mer repeats (*n* =8,386,560 comparisons) (Methods). Using similar Pearson correlation (≥0.99) and Euclidean distance cutoffs (≤5) as observed for telomeric repeat errors identified in this study (Additional file 1: Fig. S12a-c), we identified a further 2577 pairs of repeats with similar current profiles (Additional file 2: Table S1, Additional file 1: Fig. S12d). For instance, we found that (TTAGGG)_n_ telomeric repeats also showed high similarities in current profiles with repeats with single-nucleotide substitutions like (TTAAGG)_n_, (TTAGAG)_n_, and (TTGGGG)_n_ (Additional file 1: Fig. S12d,e). Repeat sequences like (GCTGCT)_n_ and (AACGGC)_n_ that differed drastically at the sequence level, but shared similar current profiles were also observed (Additional file 1: Fig. S12d,f). Further, we also examined the unmappable pool of CHM13 nanopore reads after mapping it to the CHM13 reference assembly. Remarkably, a significant pool of reads with long (GT)_n_ repeats was readily observed (Additional file 1: Fig. S13). Interestingly, (GTGTGT)_n_ repeats were also found to have high similarities in current profiles with (CTCTCT)_n_ repeats (Additional file 1: Fig. S12d, Additional file 2: Table S1), suggesting that the pool of unmappable (GT)_n_ reads may include (CT)_n_ repeats. Collectively, our results suggest that these basecalling error repeats may be observed at other repetitive regions, beyond telomeres.

It is interesting to note that telomere-like sequences are also frequently found near telomeric regions [19–22]. Specifically, there are three main types of telomere-like repeat sequences that are frequently found near telomeres in the human genome, namely the c-type repeats (TCAGGG)_n_, g-type repeats (TGAGGG)_n,_ and_,_ j-type repeats (TTGGGG)_n_ [23]. We asked if these telomere-like repeat sequences might also be basecalled incorrectly, similar to what we have observed at telomeres with (TTAGGG)_n_ repeat sequences. We therefore identified these telomere-like repeat regions from the CHM13 reference genome (Methods), and visually inspected them in IGV. These telomere-like repeat sequences could also be miscalled into repeat sequences of other repeat monomer length. For instance, we observed that the 6-mer (CCCTCA)_n_ repeats could get miscalled into the 5-mer (CCTCA)_n_ repeat sequence (Additional file 1: Fig. S14a). (CCCTGA)_n_ and (TCAGGG)_n_ 6-mer repeats could also get miscalled into (CCTGA)_n_ 5-mer repeats and (TCAGGGG)_n_ 7-mer repeats respectively (Additional file 1: Fig. S14b). Further, the 6-mer (TTGGGG)_n_ repeat was observed to be miscalled into the 7-mer (TTGGGGG)_n_ repeats (Additional file 1: Fig. S14c). We explored the current profiles for these repeats (10 consecutive repeats) using known current values for each 6-mer repeats (Additional file 1: Fig. S15). Remarkably, even though these repeat sequences were of different length, we see that these sequences can still share a highly similar current profile (Additional file 1: Fig. S15a,b,d,e,g). Of note, other 6-mer repeats were also predicted to have similar current profiles as these three types of telomere-like repeat sequences (Additional file 1: Fig. S16). Together, these suggest that the repeat miscalling errors can also be observed on these telomere-like repeat sequences. More broadly, our results also show that repeat sequences of different lengths (i.e. 6-mer vs. 5-mers and 6-mers vs. 7-mers) can share similar current profiles, and be miscalled between each other.

To see if these repeat calling errors might extend to the telomeres of other organisms, we obtained nanopore genome sequencing dataset corresponding to eight model organisms covering a wide spectrum of the tree of life from the NCBI SRA database (Fig. 1g, Additional file 2: Table S2 and S3) [24]. These eight different organisms are *Arabidopsis thaliana* [25, 26], *Caenorhabditis elegans* [27], *Gallus gallus* (chicken), *Drosophila melanogaster* [28], *Mus musculus* (mouse) [29, 30], *Saccharomyces cerevisiae* [31], *Schizosaccharomyces pombe*, and *Danio rerio* (zebrafish) [32, 33] which are all widely studied and have high-quality reference genomes. At the telomeres, these organisms are known to have (TTAGGG)_n_ telomeric repeats as humans do (chicken, zebrafish, mouse) [34–36], (TTTAGGG)_n_ repeats (*A. thaliana*) [37], (TG_1-3_)_n_ repeats (*S. cerevisiae*) [38], TTAC(A)(C)G_2-8_ (*S. pombe*) [39], (TTAGGC)_n_ repeats (*C. elegans*) [40], or retrotransposons (*D. melanogaster*) [41] (Fig. 1g). As raw current data was not available for all datasets, we directly utilized sequence data that was published by the authors of these studies. As expected, we also observed repeat calling errors on telomeres in organisms with (TTAGGG)_n_-type repeats (Additional file 1: Fig. S17a, S18), akin to what we observed in humans. Interestingly, we also observed similar telomeric repeat errors as in humans in *A. thaliana* which are known to have 7-mer (TTTAGGG)_n_ repeats (note that humans have a slightly different repeat sequence of TTAGGG) (Additional file 1: Fig. S17b, S19), which suggests that these repeats need not be 6-mer repeats (approximate number of nucleotides detected by the nanopore at each time) for errors to be observed. In *C. elegans*, (CTTGGG)_n_ repeat errors instead of (TTAGGC)_n_ telomeric repeats could also be detected in one of the two datasets assessed (Additional file 1: Fig. S17b, S20). We did not observe repeat errors for *S. cerevisiae* which are known to have (TG_1–3_)_n_ repeats at their telomeres (Additional file 1: Fig. S17c, S21), suggesting that these repeat errors do not occur on telomeres of all organisms. In organisms like *S. pombe* with more complex telomeric repeat sequences, some strand bias could be observed though we were unable to observe specific error motifs (Additional file 1: Fig. S17d). For *D. melanogaster*, which elongates telomeres via a retro-transposition-based mechanism, it was not possible to assess the frequency of repeats. Nonetheless, there was no observable difference in basecalling between the two strands at the ends of the *D. melanogaster* reference genome (Additional file 1: Fig. S22). Together, our results suggest that repeat calling errors in nanopore sequencing can be observed at telomeres of some other organisms beyond human telomeres.

**Fig. 2:**
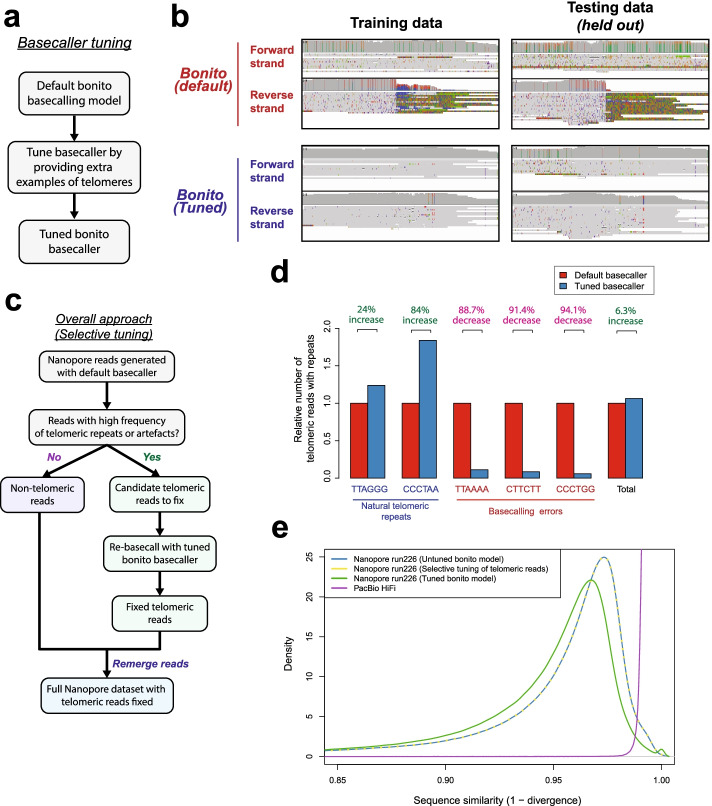
Selective re-basecalling of telomeric reads resolves basecalling errors at telomeres. **a** Approach for tuning the bonito basecalling model for improving basecalls at telomeres. **b** Tuned bonito basecalling model leads to improvement in basecalls at telomeric regions. IGV screenshots of the telomeric region (chr2q) in the CHM13 dataset basecalled using the default bonito basecaller, and the tuned bonito basecalling model is as depicted. **c** Overall approach for selecting and fixing telomeric reads in nanopore sequencing datasets. Telomeric reads are selected (“Methods”) and rebasecalled using the tuned bonito basecalling model. **d** The selective tuning approach leads to improved recovery of telomeric reads, and a decrease in the number of reads with basecalling artefacts. Evaluation was performed on the held-out test dataset (run226). **e** The “selective tuning” approach leads to little detected negative impact on basecalling of other genomic regions. The sequence similarity of all reads to the reference genome for three approaches for basecalling of nanopore reads was evaluated. They are applying the default bonito basecalling model to all reads (untuned bonito model), applying the tuned bonito basecalling model to all reads (tuned bonito model), and applying the tuned bonito basecalling model selectively to telomeric reads only (selective tuning of telomeric reads). The density plot depicts the sequence similarity of each read against the CHM13 reference genome as assessed using minimap2

To resolve these basecalling errors at telomeres, we attempted to tune the nanopore basecaller by providing it with more training examples of telomeres (Fig. 2a). Notably, model training was performed with a low learning rate to ensure that the majority of the model does not get affected during training while ensuring that minor adjustments in the model can be made to accurately basecall telomeres. Specifically, we tuned the deep neural network model underlying the Bonito basecaller by training it at a low learning rate with ground truth telomeric sequences extracted from the CHM13 reference genome, and current data of the corresponding reads (Methods). As two nanopore PromethION runs were performed on the CHM13 dataset, we used the data from one run (run225) for training and tuning of the basecaller and held out the data from the second run (run 226) for evaluation of our tuned basecaller. With this approach, we see a significant improvement in the basecalls of both the telomeres and sub-telomeric regions on the training data and held out dataset with a clearly observable decrease in errors on the chromosomal ends (Fig. 2b, Additional file 1: Fig. S23a-d).

As it is computationally more efficient to redo repeat-calling only for the small fraction of problematic telomeric reads rather than all reads, we developed an overall strategy to select these telomeric reads for re-basecalling with the tuned Bonito+telomeres basecaller (Fig. 2c). To select telomeric reads for selective re-basecalling, we relied on an observation from the CHM13 reference genome and nanopore sequencing datasets. Specifically, we noticed that telomeric reads which are mapped to the ends of the CHM13 reference genome tend to show a high frequency of telomeric, or basecalling error repeats as compared to the rest of the genome (Additional file 1: Fig. S24). We therefore utilized this observation to separate the non-telomeric reads, from the candidate telomeric reads (Fig. 2c, “Methods”). These telomeric reads were then re-base-called with the tuned Bonito basecaller before being recombined with the pool of non-telomeric reads. Remarkably, with this strategy, we observed a significant improvement in recovery of telomeric reads with (TTAGGG)_n_ and (CCCTAA)_n_ repeats (from 384 to 476 TTAGGG and 373 to 686 CCCTAA reads) (Fig. 2d). At the same time, a sharp reduction of these basecalling repeat errors was also observed (151 to 17 TTAAAA reads, 561 to 48 CTTCTT reads, and 337 to 20 CCCTGG reads) (Fig. 2d). Our “selective tuning” approach for fixing basecalling errors at telomeres can improve recovery of telomeric reads while reducing telomeric basecalling repeat artefacts.

We further evaluated our approach for possible impact on overall basecalling accuracy. While a reduction in global basecalling accuracy was observed (~1–2%) when our tuned basecaller was directly applied to the full dataset, caused likely by miscalling of endogenous (CTTCTT)_n_ genomic repeats as (CCCTAA)_n_, this loss of global basecalling accuracy could be avoided by applying our basecaller to telomeric reads alone. Concordant with this, we did not observe changes in overall basecalling accuracy with our telomere-selective tuning approach (Fig. 2e). These results indicate that our telomere-selective tuning approach has a negligible impact on basecalling accuracy for the rest of the genome.

## Conclusion

In this study, we showed that basecalling errors can be widely observed at telomeric regions across nanopore datasets, sequencing platforms, basecallers, and basecalling models. These repeat errors further extend to telomeres of other organisms with (TTAGGG)_n_ repeats, to organisms with non-(TTAGGG)_n_ repeats, and also to repeats with different monomer length. We further showed that these strand-specific basecalling errors were likely induced by similarities in current profiles between different repeat types. To resolve these basecalling errors at telomeres, we devised an overall strategy to re-basecall telomeric reads using a tuned nanopore basecaller. More broadly, our study highlights the importance of verifying nanopore basecalls in long, repetitive, and poorly defined regions of the genome. For instance, this can be done either with an orthogonal platform or at a minimum by ensuring nanopore basecalls between opposite strands are concordant. An extensive evaluation of genome-wide basecalling errors in repeat regions is also needed in the future given our observations at telomeric regions. Nonetheless, we anticipate that subsequent further improvements in the nanopore basecaller or basecalling model as demonstrated in this study will potentially lead to the reduction or elimination of these basecalling artefacts.

## Methods

### Nanopore and PacBio datasets

Nanopore and PacBio HiFi datasets for the CHM13 sample were downloaded directly from the telomere-to-telomere consortium (https://github.com/marbl/CHM13) [14, 15].

Nanopore dataset for GM12878 was obtained from the Nanopore WGS consortium (https://github.com/nanopore-wgs-consortium/NA12878/blob/master/Genome.md) [13]. PacBio HiFi dataset for GM12878 was obtained from the repository at the SRA database (SRP194450) [17, 18] and downloaded from the following link (https://www.ebi.ac.uk/ena/browser/view/SRR9001768?show=reads).

The HG002 PacBio HiFi and Nanopore datasets were downloaded from the Human Pangenome Reference Consortium (https://github.com/human-pangenomics/HG002_Data_Freeze_v1.0) [12]. Specifically, the HG002 Data Freeze (v1.0) recommended downsampled data mix was downloaded. The PacBio HiFi dataset corresponds to ~34× coverage of Sequel II System with Chemistry 2.0. The nanopore dataset corresponds to 60x coverage of unsheared sequencing from 3 PromethION flow cells from Shafin et al [42].

### Extraction of candidate telomeric reads

Telomeric reads were extracted by mapping all reads to the CHM13 draft genome assembly (v1.0) obtained from the telomere-to-telomere consortium using Minimap2 (version 2.17-r941) [43]. Subsequent to that, reads that mapped to within 10 kilobase pairs of the start and end of each autosome and X-chromosome were then extracted using SAMtools (version 1.10) [44].

### Co-occurrence matrix

Candidate PacBio HiFi and Nanopore telomeric reads were first extracted as described above and then converted into the FASTA format using SAMtools (version 1.10) [44]. Subsequent to that, custom Python scripts were used to assess if each of the reads contain at least four consecutive counts of the repeat sequence of interest (e.g. (TTAGGG)_4_). This information is then used to generate a pair-wise correlation matrix as depicted with R in the main text.

### Basecalling of nanopore data with different basecallers and basecalling models

Basecalling of nanopore data was done using Guppy (Version 4.4.2), Guppy (Version 5.0.16) and Bonito v0.3.5 (commit d8ae5eeb834d4fa05b441dc8f034ee04cb704c69). For Guppy4, four different basecalling models were applied (guppy_dna_r9.4.1_450bps_fast, guppy_dna_r9.4.1_450bps_hac, guppy_dna_r9.4.1_450bps_prom_fast, guppy_dna_r9.4.1_450bps_prom_hac). For Guppy 5, six different basecalling models were applied (dna_r9.4.1_450bps_fast, dna_r9.4.1_450bps_hac, dna_r9.4.1_450bps_sup, dna_r9.4.1_450bps_fast_prom, dna_r9.4.1_450bps_hac_prom, dna_r9.4.1_450bps_sup_prom)

For Bonito, the v1, v2, v3, v3.1, and default basecalling models were applied.

### Current profiles for different repeat sequences

The mean current level for different k-mers sequenced by nanopore sequencing was obtained from the k-mer models published by Oxford Nanopore (https://github.com/nanoporetech/kmer_models/tree/master/r9.4_180mv_450bps_6mer). Circular permutations of each 6-mer of interest were generated, and their corresponding mean current level was extracted from the k-mer models. The current profiles for each of the indicated repeat sequences were then plotted and depicted in the figure.

### Pairwise comparison of all possible k-mers

Current profile for each 6-mer repeat sequence was generated using the published k-mer models as described above. Pairwise comparisons of all possible 6-mer repeat current profiles were then performed (8,386,560 pairs in total). A corresponding (i) Pearson correlation value, (ii) mean-centered Euclidean distance, and (iii) mean current difference for each pair of 6-mer repeat current profiles were then generated. Pairs of repeats with a Pearson correlation value ≥ 0.99 and Euclidean distance ≤ 5 were selected as putative repeat pairs that can be miscalled.

### Tuning of bonito model

The default model from Bonito v0.3.5 (commit d8ae5eeb834d4fa05b441dc8f034ee04cb704c69) was used as the base model for model tuning. The training dataset needed for the training process was generated from the telomeric reads from a PromethION run in the CHM13 dataset (run225). More broadly, we then generate the training dataset by matching the current profiles from the nanopore run to ground truth sequences that we extracted from the CHM13 draft reference genome assembly (v1.0) using custom written code.

Specifically, these telomeric reads were first basecalled using the initial Bonito basecalling model and then mapped back to the CHM13 draft reference genome assembly (v1.0). This allowed each telomeric read to be properly assigned to its corresponding chromosomal arm with its sub-telomeric sequence. Nonetheless, as the telomeric region of the same read could not be properly mapped to the telomeric repeats due to the repeat errors, there was difficulty in assigning the nanopore current data to the correct ground truth sequences in the reference genome. As such, the presumed length of sequences to extract was estimated using the basecalling repeat error sequences, and the same length of sequences was then extracted from the CHM13 reference genome to serve as ground truth sequences. With this idea and with a custom Perl script, we were able to generate a set of ground truth sequences and signals for model tuning. These data were then formatted into the corresponding Python objects required by the Bonito basecaller with custom Python scripts. Using the tune function in Bonito and with our prepared training dataset, we were then able to train the basecaller to convergence.

### Selective application of tuned basecaller to telomeric reads

We applied our tuned basecaller by first extracting candidate telomeric reads for re-basecalling. This was done by enumerating the total 3-mer telomeric (i.e., (TTAGGG)_3_, (CCCTAA)_3_) and repeat artefact count (i.e. (TTAAAA)_3_, (CTTCTT)_3_, (CCCTGG)_3_) on each read. Reads with at least 10 total counts of these repeats were isolated and their readnames noted. These reads were then excluded from the total pool of reads via their readnames, and basecalled separately using our tuned basecaller using the fast5 data of these reads. Following basecalling with the tuned basecaller, these reads were then recombined with the main pool of reads.

### Evaluation of repeat calling errors in model organisms

Nanopore genome sequencing (and where available PacBio HiFi) datasets corresponding to eight model organisms were identified from the NCBI SRA database [24]. A full list of the datasets used in this study is as indicated in Additional file 2: Table S2. Specifically, runs for each of the following organisms were analyzed: *A. thaliana* (SRR14474199, SRR16149191) [25, 26], *C. elegans* (SRR15993157, SRR16936857) [27], Chicken (SRR15420785, SRR15420786, SRR15420787, SRR15421342 to SRR15421346), *D. melanogaster* (SRR15107931 to SRR15107934, SRR15107937) [28], Mouse (SRR11606870, SRR14685232, SRR14685224 to SRR14685243) [29, 30], *S. cerevisiae* (ERR6318522, ERR6318523) [31], *S. pombe* (SRR17382753, SRR18210325), and Zebrafish (SRR17257555, SRR15037325) [32, 33].

The corresponding fastq files for each of these runs were then downloaded from the SRA database and then mapped to their corresponding reference genomes using minimap2 with the parameter -x map-ont. The reference genomes used for read mapping of each of the organisms are as follows: *A. thaliana* (TAIR10), *C. elegans* (ce11), Chicken (galGal6), *D. melanogaster* (dm6), Mouse (mm39), *S. cerevisiae* (sacCer3), *S. pombe* (https://www.pombase.org/data/genome_sequence_and_features/genome_sequence/Schizosaccharomyces_pombe_all_chromosomes.fa.gz), and Zebrafish (danRer11). Alignments of these nanopore datasets for each of these organisms were then visualized together with their corresponding reference genomes in IGV at the annotated terminal ends. Note that as not all chromosomal ends were well assembled in these organisms, only selected chromosomal arms could be readily visualized and inspected in IGV for the presence of these repeat calling errors. To generate plots summarizing the frequency of telomeric repeats and repeat errors in each organism, reads on the terminal 10kb region of each chromosomal arms were extracted. The only exception was mouse in which the terminal 500kb region of the reference genome was extracted as the ends of the reference genome were padded by very long stretches of NNNs.

### Sequencing coverage of each chromosomal arms

The number of reads at 10kb, 100kb, and 1000kb of the annotated ends at each chromosomal arm in the CHM13 reference was extracted using SAMtools [44] and then counted. Boxplot corresponding to the distribution of reads observed on each arm was then generated using R.

### Simulation of long-reads to assess mismapping rates at sub-telomeres in CHM13

PBSIM2 [45] was used to simulate long-reads from the CHM13 reference genome with the parameters --depth 100 --length-min 5000 --length-mean 10000 --accuracy-mean 0.95 --hmm_model R94.model. In some instances, we also modified the read accuracy from 0.95 to 0.98 or to 0.999 to assess the impact of the read accuracy on the mismapping rate. The pbsim2fq function in the paftools.js script (distributed as part of minimap2) [43] was then used to generate fastq files with readnames corresponding to the true read positions from the .maf files from PBSIM2. Reads that originated from the terminal 1000kb, 100kb, or 10kb region of the CHM13 reference genome (i.e., overlap with these regions with least one base-pair) were then extracted and then mapped to the CHM13 reference genome using minimap2 (version 2.17-r941) [43]. The mapeval function in the paftools.js script was then used to evaluate the accuracy of read mapping of reads extracted from these regions.

### Evaluation of errors at telomere-like repeat regions

To evaluate the presence of repeat calling errors at telomere-like regions, we first identified regions in the CHM13 reference genome with telomere-like repeat sequences. This was done by mapping the CHM13 reference to an artificial reference containing 600 repeats of each of the three types of telomere-like repeats. In all, we identified 7 regions with (TCAGGG)_n_, 7 regions with (TGAGGG)_n_, and 5 regions with (TTGGGG)_n_ repeats that are at least 100 bp in length. Each of these regions was then manually inspected in IGV for the occurrence of these repeat errors.

### Evaluation of more error-prone and less error-prone reads

To establish which reads are more error-prone or less error-prone, we calculated the number of non-overlapping telomeric repeats ((TTAGGG)_3_ and (CCCTAA)_3_) and artefact repeats ((TTAAAA)_3_, (CCCTGG)_3_, and (CTTCTT)_3_) on each long-read using custom Python scripts. The proportion of repeat errors on each long-read was then calculated by dividing the number of artefact repeats on each long-read with the total number of telomeric and artefact repeats.

### Current profile plots

Raw current values were extracted from fast5 files using the h5py package in Python. The raw current values were then converted to actual current values using the formula: current_in_pA = scale * (raw_current_value + offset), where the offset was extracted directly from the fast5 file, and the scale was calculated as scale = range/digitization. The range and digitization values were extracted directly from the metadata of the fast5 files. Current profiles were then visualized as depicted.

## Supplementary Information

Additional file 1: Figure S1. Additional screenshots of basecalling repeat errors found on different chromosomal arms. Figure S2. Examples of long-reads with three types of basecalling error repeats found at telomeres. Figure S3. Co-occurrence heatmap illustrating the frequency of co-occurrence of telomeric repeats and basecalling errors for the CHM13 nanopore dataset generated at different sites. Figure S4. Frequency of telomeric repeats and repeat artefacts on each chromosomal arm. Figure S5. Mapping error rate of long-reads simulated from terminal ends of the CHM13 reference genome. Figure S6. Negligible bias in read coverage of each of the chromosomal arms was observed in the nanopore sequencing dataset for the CHM13 sample. Figure S7. Frequency of telomeric repeat errors in different nanopore sequencing dataset and sequencing platforms. Figure S8. Frequency of telomeric repeat errors in different nanopore basecallers. Figure S9. Co-occurrence heatmap for different nanopore basecalling models. Figure S10. Frequency of telomeric repeats and repeat artefacts on each long read. Figure S11. Current profiles for telomeric repeats in reads of low read qualities, or in reads of high read qualities. Figure S12. Similarities between current profiles for all possible pairs of 6-mer repeats. Figure S13. Example of reads with (GT)n repeat sequences in the CHM13 dataset. Figure S14. IGV screenshots depicting repeat calling errors observed on telomere-like repeat sequences in the CHM13 dataset. Figure S15. Simulated current profiles for 10 consecutive repeats of the telomere-like repeat sequences, and observed repeat calling errors. Figure S16. Repeats with predicted similarity in current profiles to the three types of telomere-like repeat sequences. Figure S17. Frequency of natural telomeric repeats, and repeat calling errors in nanopore datasets for each organism assessed. Figure S18. Repeat calling errors are present on the telomeres of Chicken which are characterized by (TTAGGG)n repeat sequences. Figure S19. Repeat calling errors are present on the telomeres of Arabidopsis thaliana which are characterized by (TTTAGGG)n repeat sequences. Figure S20. Repeat calling errors are present on the telomeres of some Caenorhabditis elegans nanopore datasets. C. elegans telomeres are characterized by (TTAGGC)n repeat sequences. Figure S21. Repeat calling errors are absent on the telomeres of Saccharomyces cerevisiae which are characterized by (TG1–3)n repeat sequences. Figure S22. No differences in basecalling was observed between different strands at the terminal end of Drosophila melanogaster. Figure S23. Additional examples for the performance of the tuned bonito basecaller on telomeres on other chromosomal arms. Figure S24. Histograms depicting the frequencies of 3-mer repeats on reads at telomeres and on reads found at the rest of the genome in the CHM13 dataset.

Additional file 2: Table S1. List of k-mers with high similarities in current profiles. The pearson correlation, Euclidean distance, and mean current difference between each pair of k-mer is as presented in the table. Table S2. List of nanopore datasets from different organisms utilized in this study. The accession number for each of the runs, the nanopore platform used for generating the dataset, and where available the basecaller used for basecalling are as indicated. Table S3. Telomeric repeats for organisms assessed and links to publications supporting these telomeric repeats.

Additional file 3: Review history

## Data Availability

Source code to apply and retrain the bonito bascalling model for telomeric region can be found at the following link: https://github.com/ktan8/nanopore_telomere_basecall/ [46] and also on Zenodo [47]. The tuned bonito basecalling model can be downloaded from https://zenodo.org/record/6982661/files/chm13_nanopore_trained_run225.zip [47]. A comprehensive version of Additional file 2: Table S1 with all possible pairs of k-mers can be found at https://zenodo.org/record/6982661/files/all_comparisions.similar_profile.txt.zip [47]. Details on the Human Nanopore and PacBio Datasets used for this study are available in the sub-section on *Nanopore and PacBio Datasets* in the Methods Section [12–15, 17, 18, 42]. The full list of Nanopore and PacBio datasets for other organisms analyzed in this study is available in Additional file 2: Table S2 and were obtained from the corresponding publications [25–33], though we would also like to note that some datasets we had obtained from the SRA database do not have a corresponding publication.
