# Supplementary material for: Identifying and correcting repeat-calling errors in nanopore sequencing of telomeres
Source: Genome Biol. 2022 Aug 26;23:180. doi: 10.1186/s13059-022-02751-6 (PMC9414165; doi:10.1186/s13059-022-02751-6)
Supplement: Supplementary file 1 — Additional file 1: Figure S1. Additional screenshots of basecalling repeat errors found on different chromosomal arms. Figure S2. Examples of long-reads with three types of basecalling error repeats found at telomeres. Figure S3. Co-occurrence heatmap illustrating the frequency of co-occurrence of telomeric repeats and basecalling errors for the CHM13 nanopore dataset generated at different sites. Figure S4. Frequency of telomeric repeats and repeat artefacts on each chromosomal arm. Figure S5. Mapping error rate of long-reads simulated from terminal ends of the CHM13 reference genome. Figure S6. Negligible bias in read coverage of each of the chromosomal arms was observed in the nanopore sequencing dataset for the CHM13 sample. Figure S7. Frequency of telomeric repeat errors in different nanopore sequencing dataset and sequencing platforms. Figure S8. Frequency of telomeric repeat errors in different nanopore basecallers. Figure S9. Co-occurrence heatmap for different nanopore basecalling models. Figure S10. Frequency of telomeric repeats and repeat artefacts on each long read. Figure S11. Current profiles for telomeric repeats in reads of low read qualities, or in reads of high read qualities. Figure S12. Similarities between current profiles for all possible pairs of 6-mer repeats. Figure S13. Example of reads with (GT)n repeat sequences in the CHM13 dataset. Figure S14. IGV screenshots depicting repeat calling errors observed on telomere-like repeat sequences in the CHM13 dataset. Figure S15. Simulated current profiles for 10 consecutive repeats of the telomere-like repeat sequences, and observed repeat calling errors. Figure S16. Repeats with predicted similarity in current profiles to the three types of telomere-like repeat sequences. Figure S17. Frequency of natural telomeric repeats, and repeat calling errors in nanopore datasets for each organism assessed. Figure S18. Repeat calling errors are present on the telomeres of Chicken which are characterized by ( [file 13059_2022_2751_MOESM1_ESM.docx]

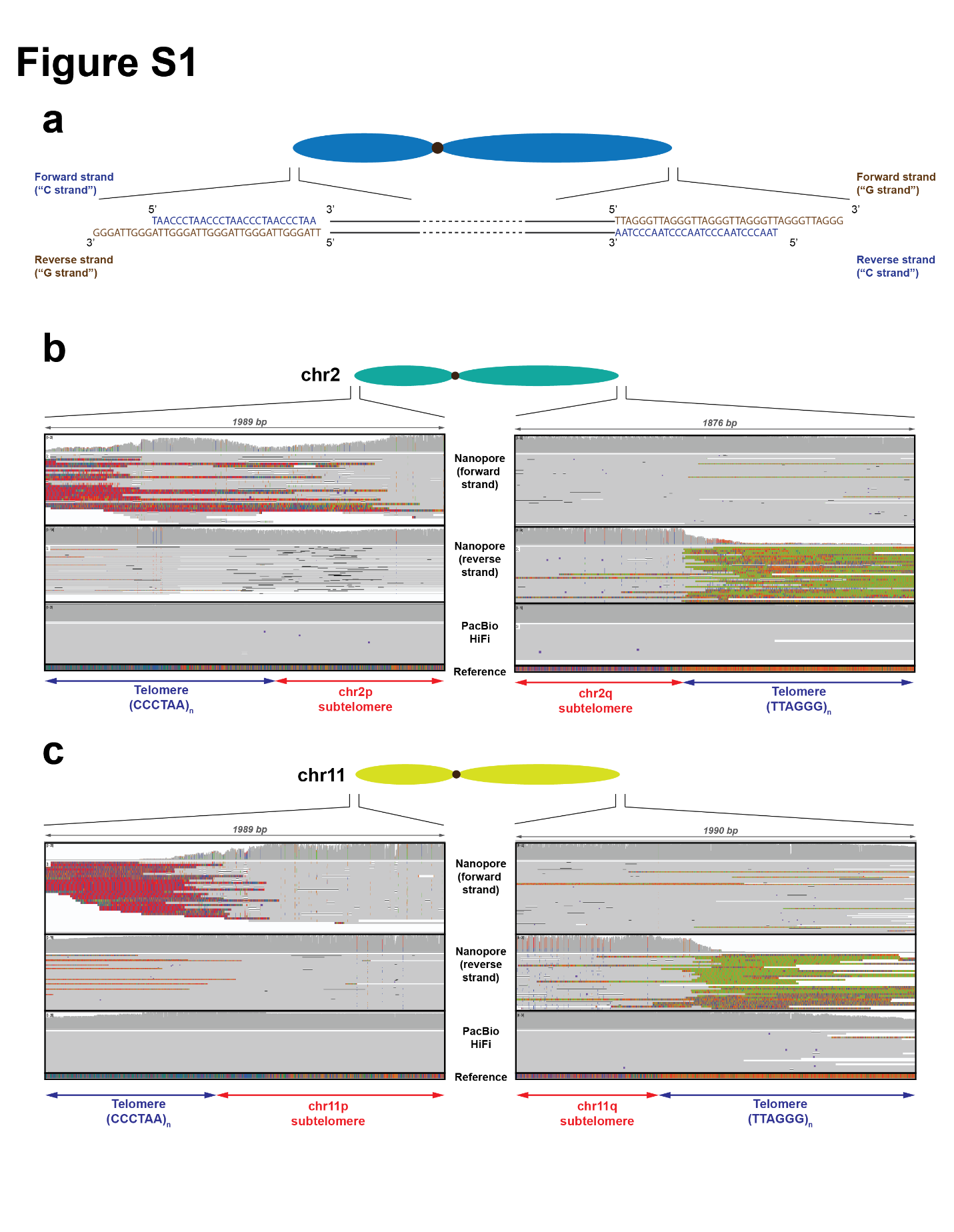


**Fig. S1 Additional screenshots of basecalling repeat errors found on different chromosomal arms.** **(a)** Schematic depicting sequence and orientation of telomeric repeat sequences on the p-arms (arm on the left in the schematic) and q-arms (arm on the right of the schematic) of a chromosome. Note that the forward strand for the arm on the left, and reverse strand for the arm on the right are “C-rich strands” and characterized by (CCCTAA)_n_ repeats in a 5’-to-3’ direction. Also note that the reverse strand for the arm on the left, and forward strand for the arm on the right are “G-rich strands” and characterized (TTAGGG)_n_ repeats in a 5’-to-3’ direction. **(b-c)** Screenshots depicting additional representative examples of chromosomal arms with basecalling error repeats. These are **(b)** chromosome 2 and **(c)** chromosome 11. Screenshots were extracted from the Integrative Genomics Viewer for the CHM13 long-read dataset mapped against the CHM13 reference genome. Related to Figure 1a.


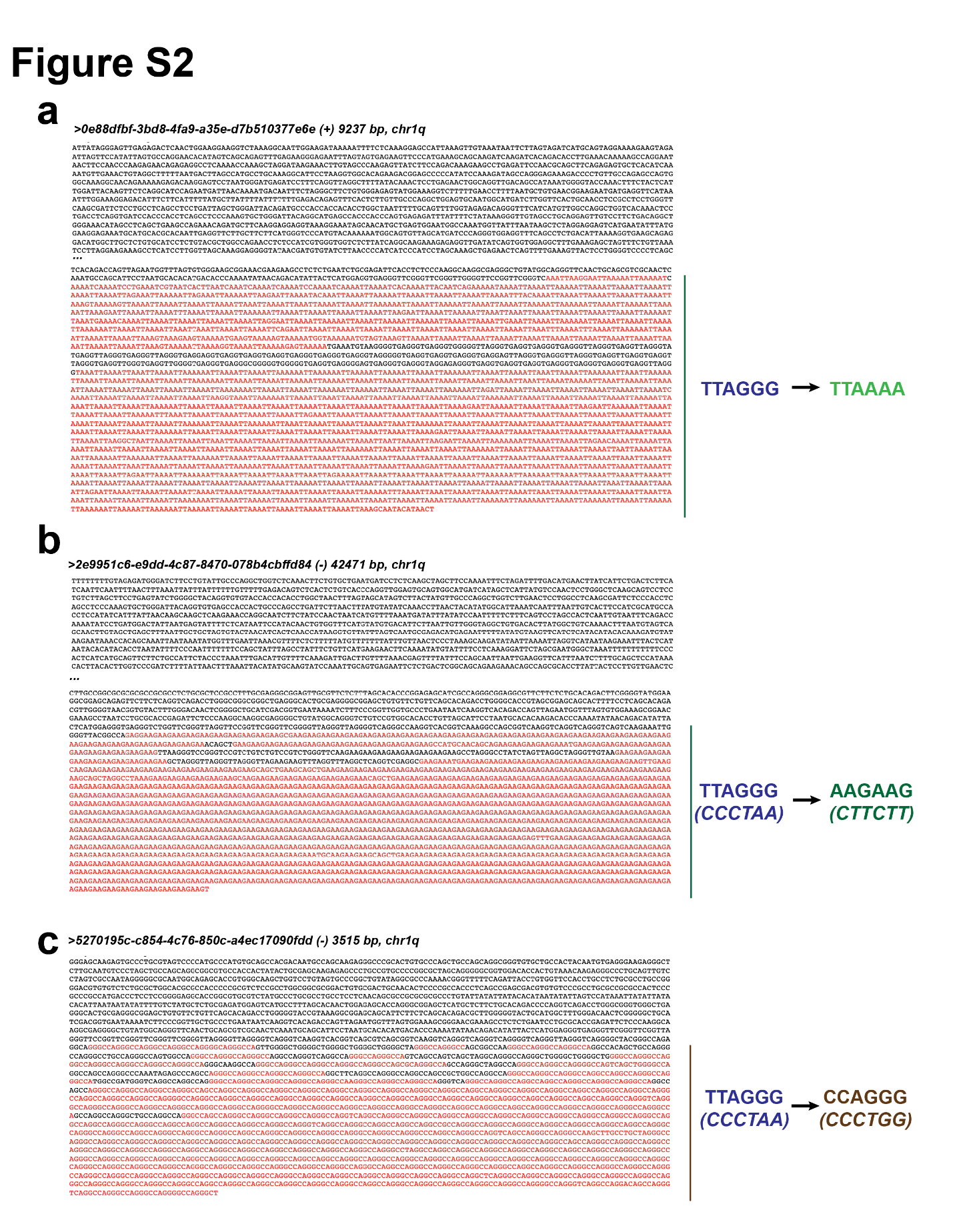
 **Fig. S2 Examples of long-reads with three types of basecalling error repeats found at telomeres. (a-c)** Sequences and readnames of representative long-reads with the three reported types of basecalling error repeats are as depicted. The region with the basecalling error repeats is highlighted in red. The three type of basecalling errors found on each long read are **(a)** (TTAGGG)_n_ to (TTAAAA)_n_, **(b)** (CCCTAA)_n_ to (CTTCTT)_n_ and **(c)** (CCCTAA)_n_ to (CCCTGG)_n_. Note that **(b)** and **(c)** represents the reverse complementary sequence of the actual nanopore long-read sequence. Also note that the repeats were found on the end of each read as expected given that telomeric repeats are typically found on the end of the chromosomes.


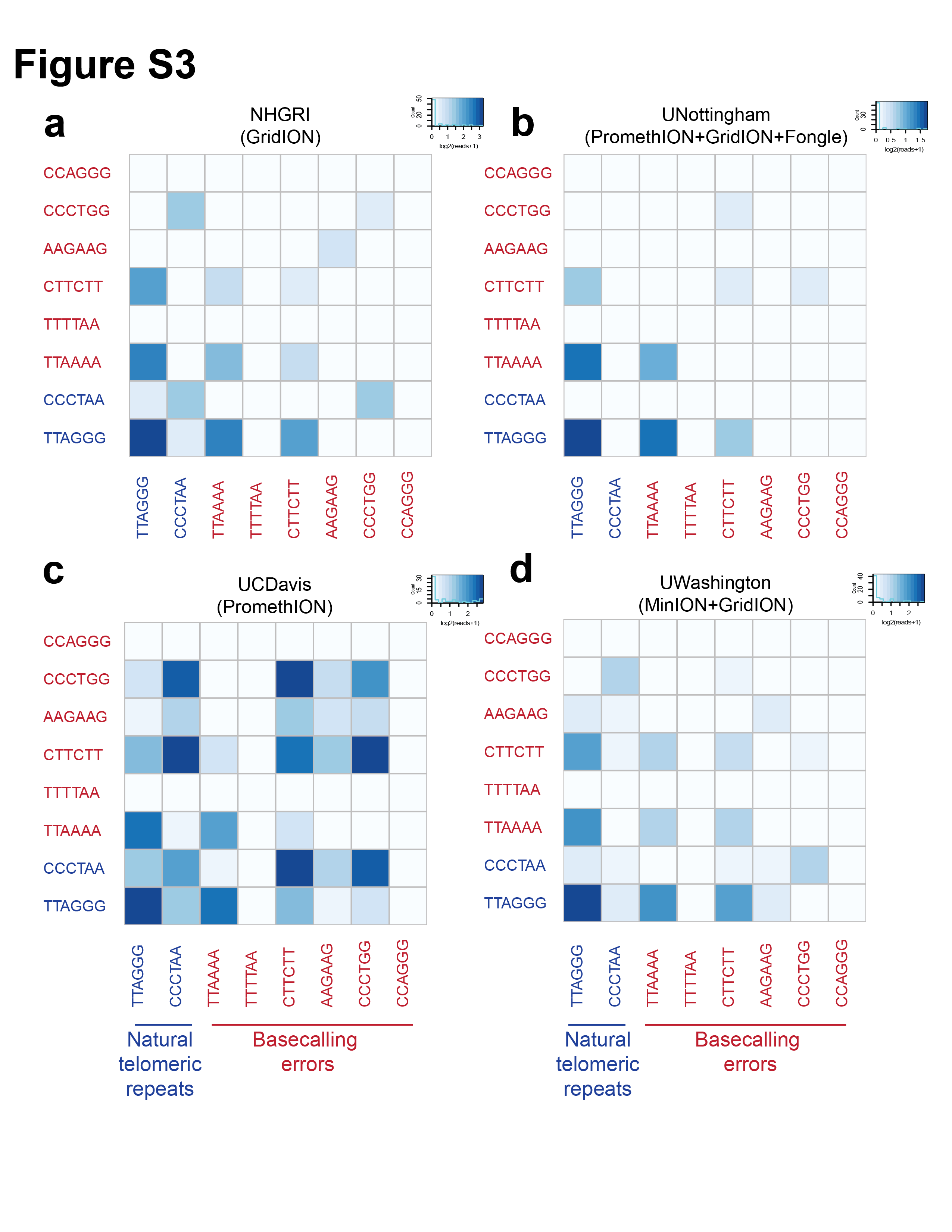
 **Fig. S3 Co-occurrence heatmap illustrating the frequency of co-occurrence of telomeric repeats and basecalling errors for the CHM13 nanopore dataset generated at different sites.** These are **(a)** National Human Genome Research Institute (NHGRI), **(b)** University of Nottingham (UNottingham), **(c)** University of California, Davis (UCDavis) and **(d)** University of Washington (UWashington). The sequencing platforms used for sequencing at each of the sites are also as indicated. This figure is related to Figure 1c.


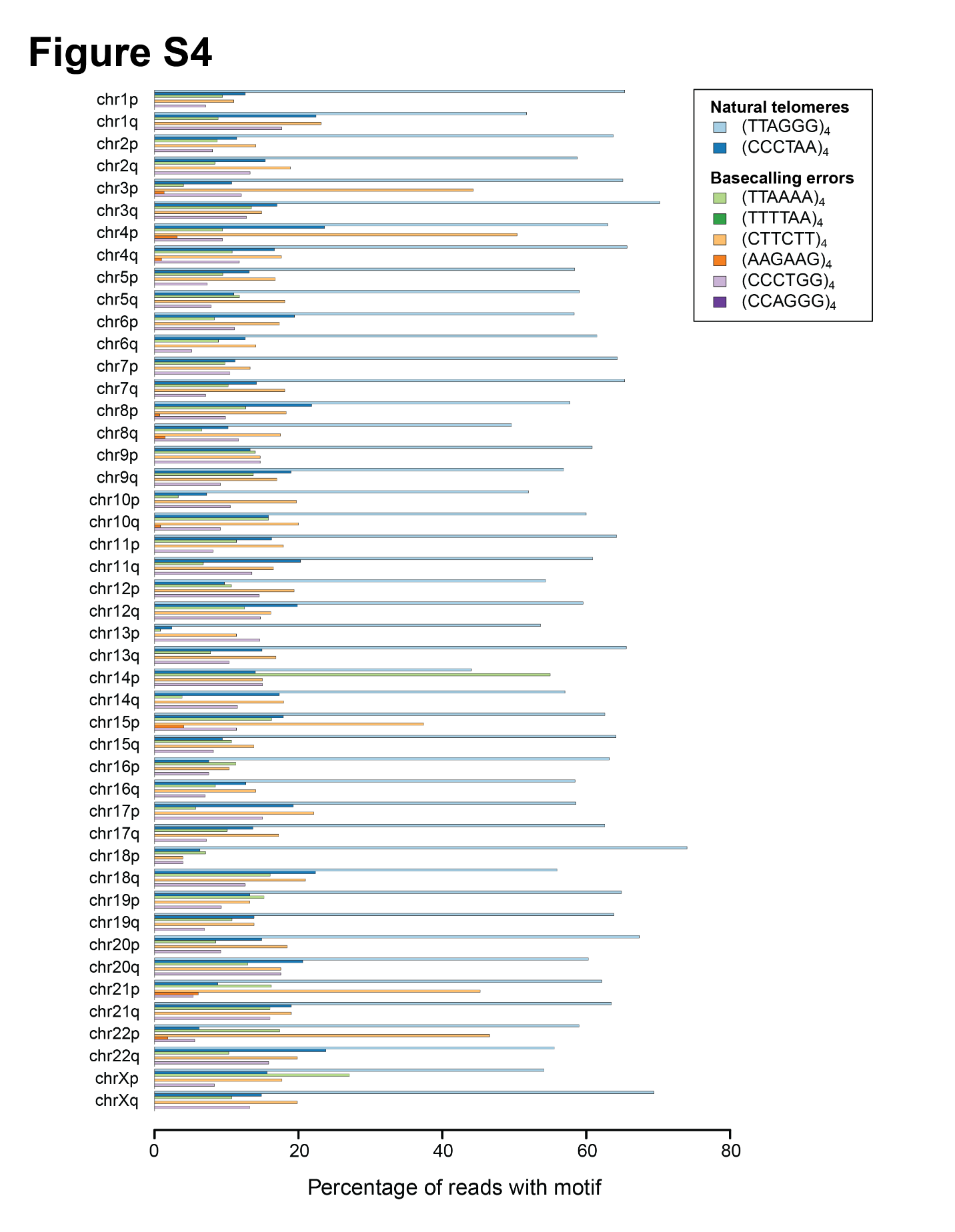


**Fig. S4 Frequency of telomeric repeats and repeat artefacts on each chromosomal arm.** Frequency of telomeric repeats, and repeat errors on each chromosomal arm in the CHM13 dataset as depicted. Nanopore reads presented in this plot were basecalled using the Bonito basecaller.


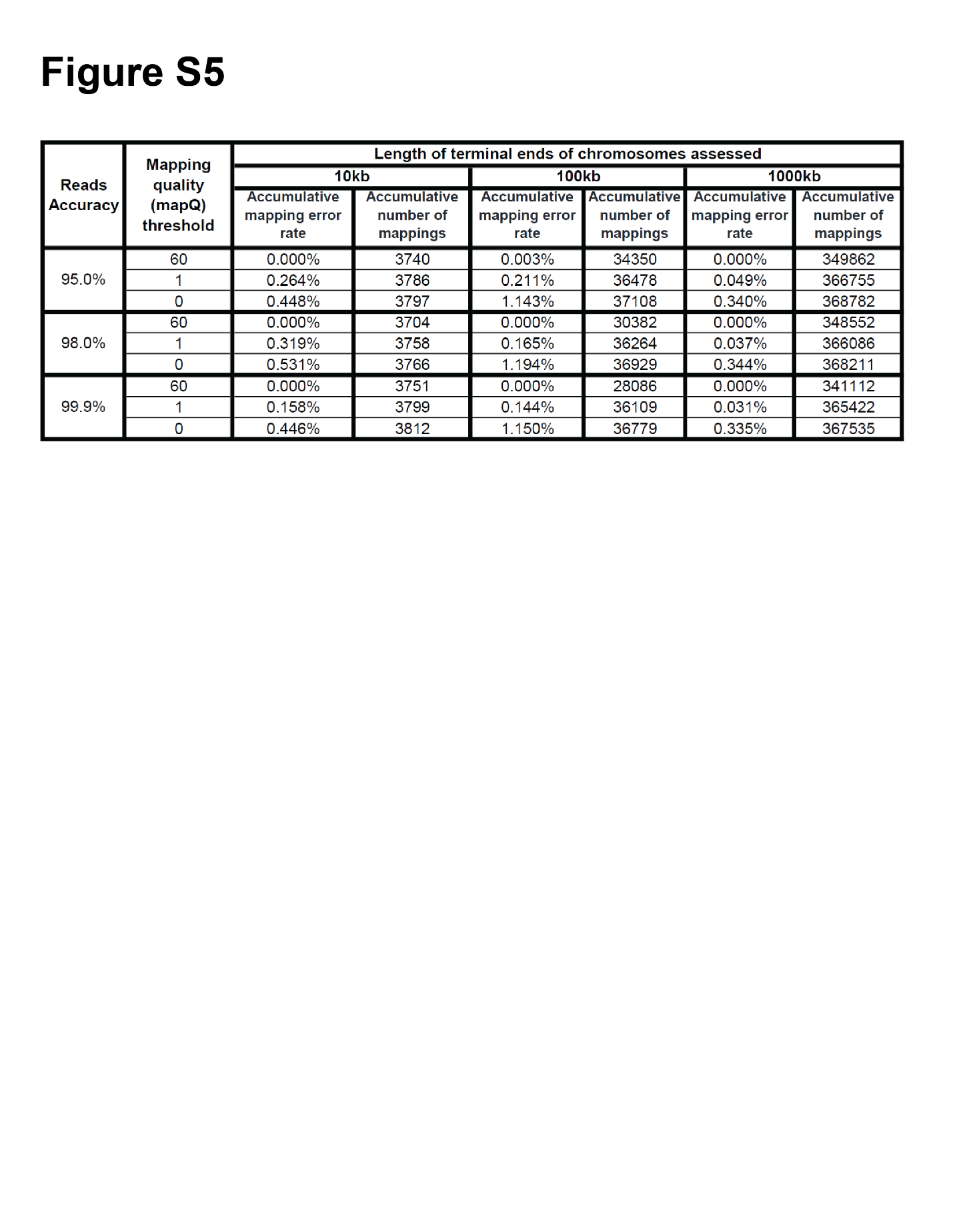


**Fig. S5 Mapping error rate of long-reads simulated from terminal ends of the CHM13 reference genome.** Long-reads were simulated from the terminal 10kb, 100kb or 1000kb ends of each chromosome in the CHM13 reference genome. Different error rates for the reads was also assessed, with read accuracy of 95.0%, 98.0%, and 99.9%. Reads were then remapped to the CHM13 reference genome with minimap2 and the corresponding mis-mapping rate of these reads assessed under different mapping quality thresholds.


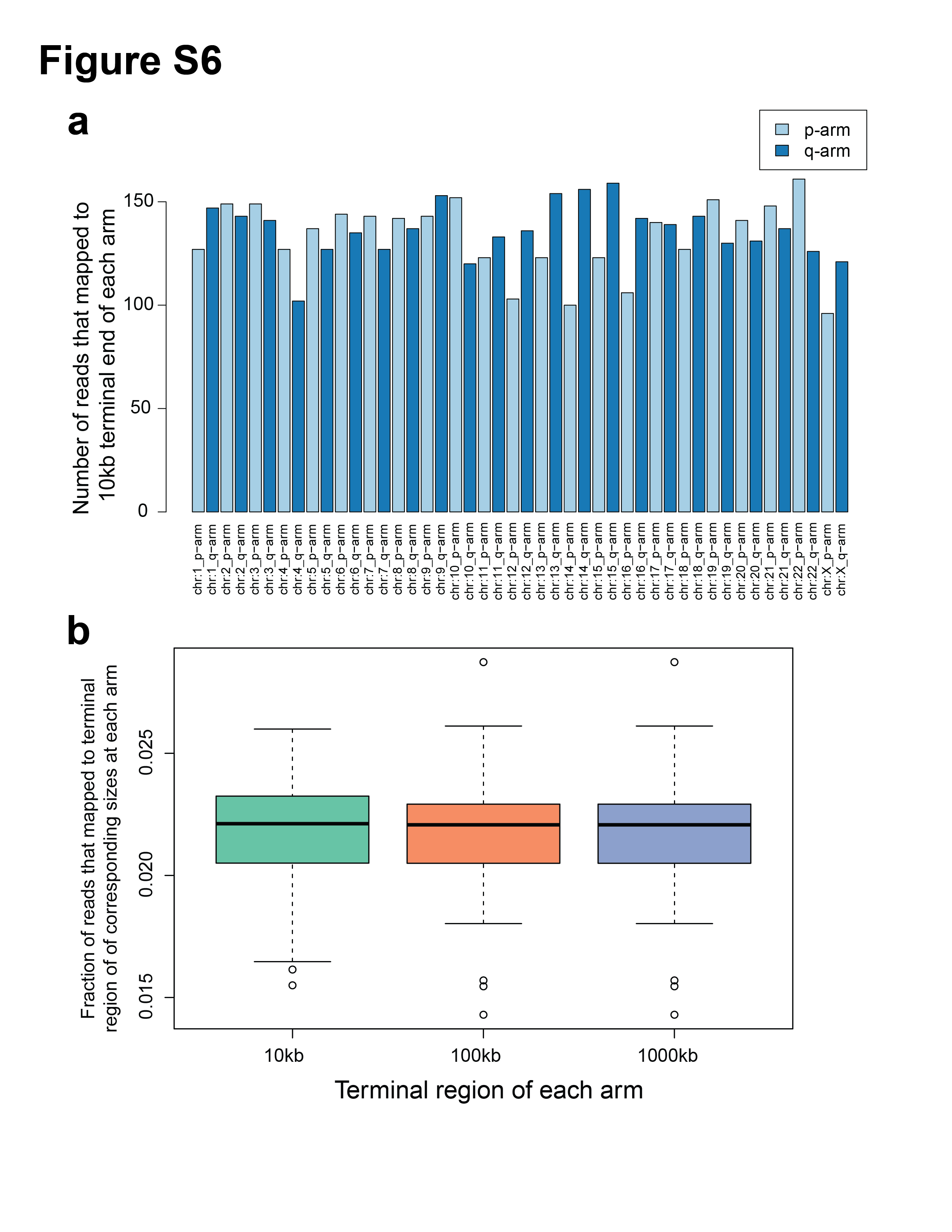
 **Fig. S6 Negligible bias in read coverage of each of the chromosomal arms was observed in the nanopore sequencing dataset for the CHM13 sample. (a)** Barplot depicting the number of reads mapped to the flanking 10kb region of each chromosomal arm of the CHM13 reference genome. **(b)** Boxplot depicting the fraction of reads that mapped to each chromosomal arm. The fraction of reads mapped to each arm was calculated by dividing the number of reads observed at each of the arm by the total number of reads across all chromosomal arms for the corresponding terminal region sizes of 10kb, 100kb, or 1000kb. Similar levels of variation were observed for reads mapped to the terminal 10kb region where most mapping errors are expected, as the terminal 1000kb region where mapping errors are less likely to occur.


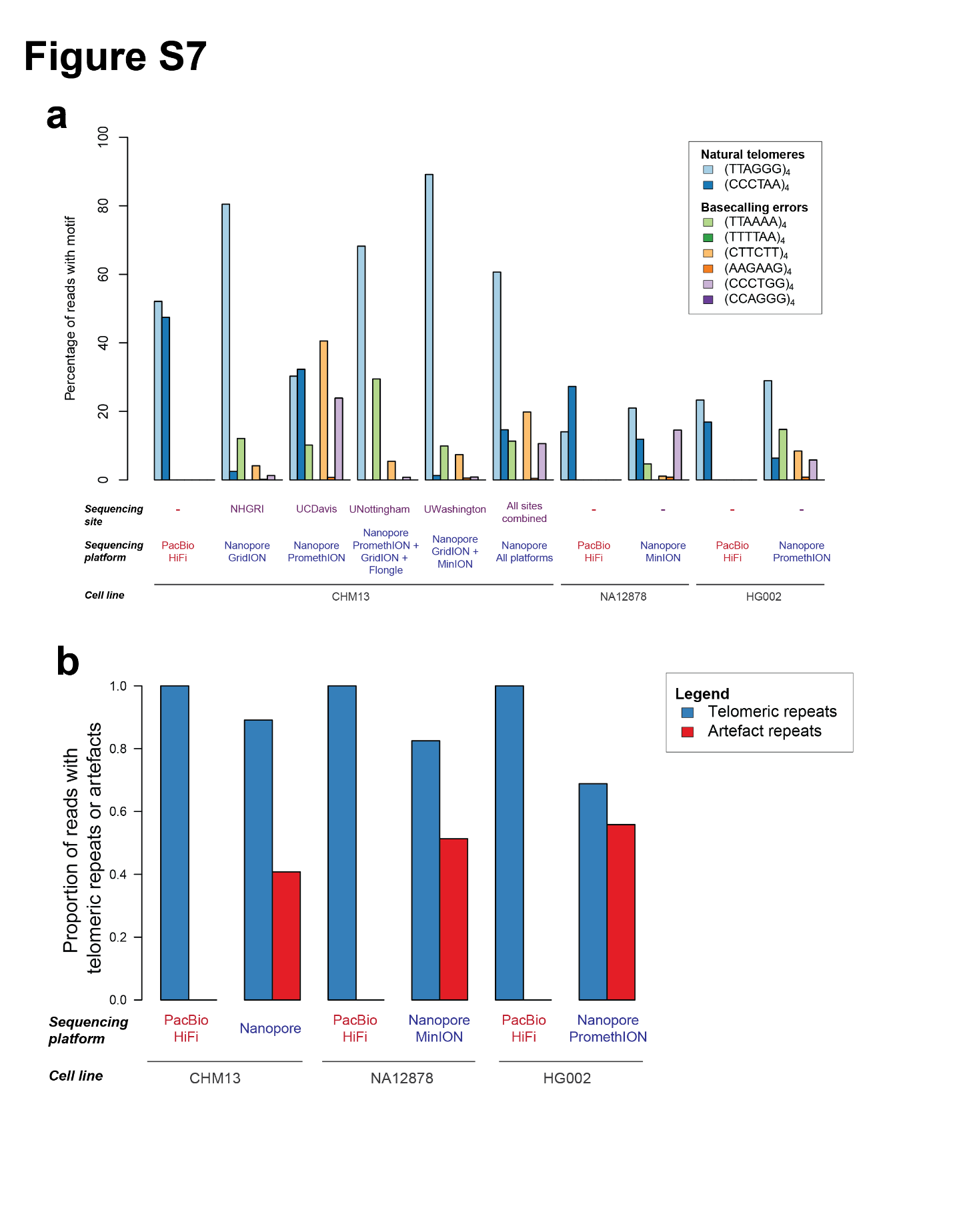


**Fig. S7 Frequency of telomeric repeat errors in different nanopore sequencing dataset and sequencing platforms. (a)** Frequency of basecalling error repeats in three different cell lines generated by different nanopore sequencing platforms. This figure is an extension Figure 1d. **(b)** Aggregated fraction of basecalling error repeats for different cell lines and datasets.


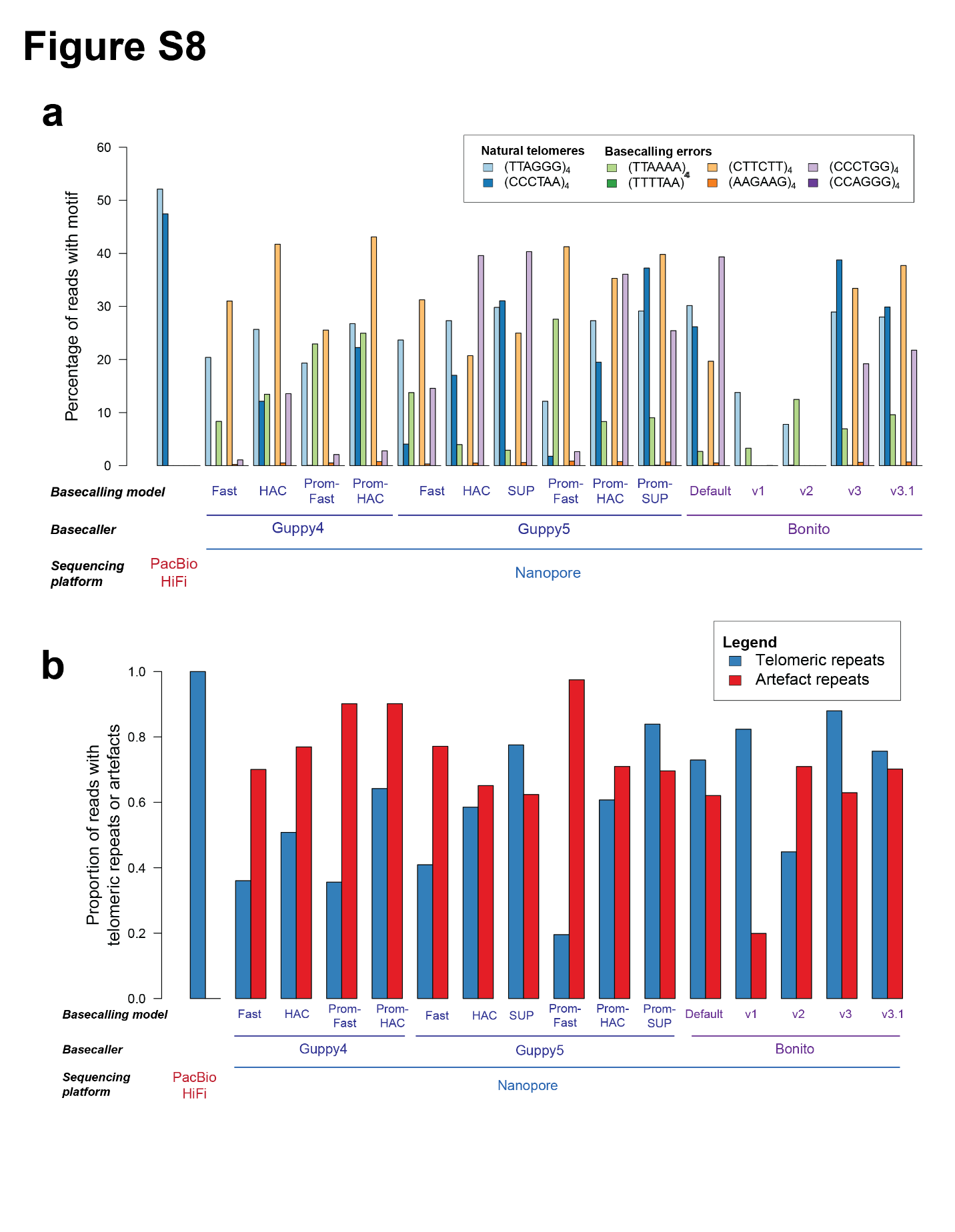
**Fig. S8 Frequency of telomeric repeat errors in different nanopore basecallers. (a)** Frequency of basecalling error repeats for different basecallers (Guppy4, Guppy5 and Bonito) and basecalling models. This figure is an extension of Figure 1e. **(b)** Aggregated fraction of basecalling error repeats for different basecallers and basecalling models.


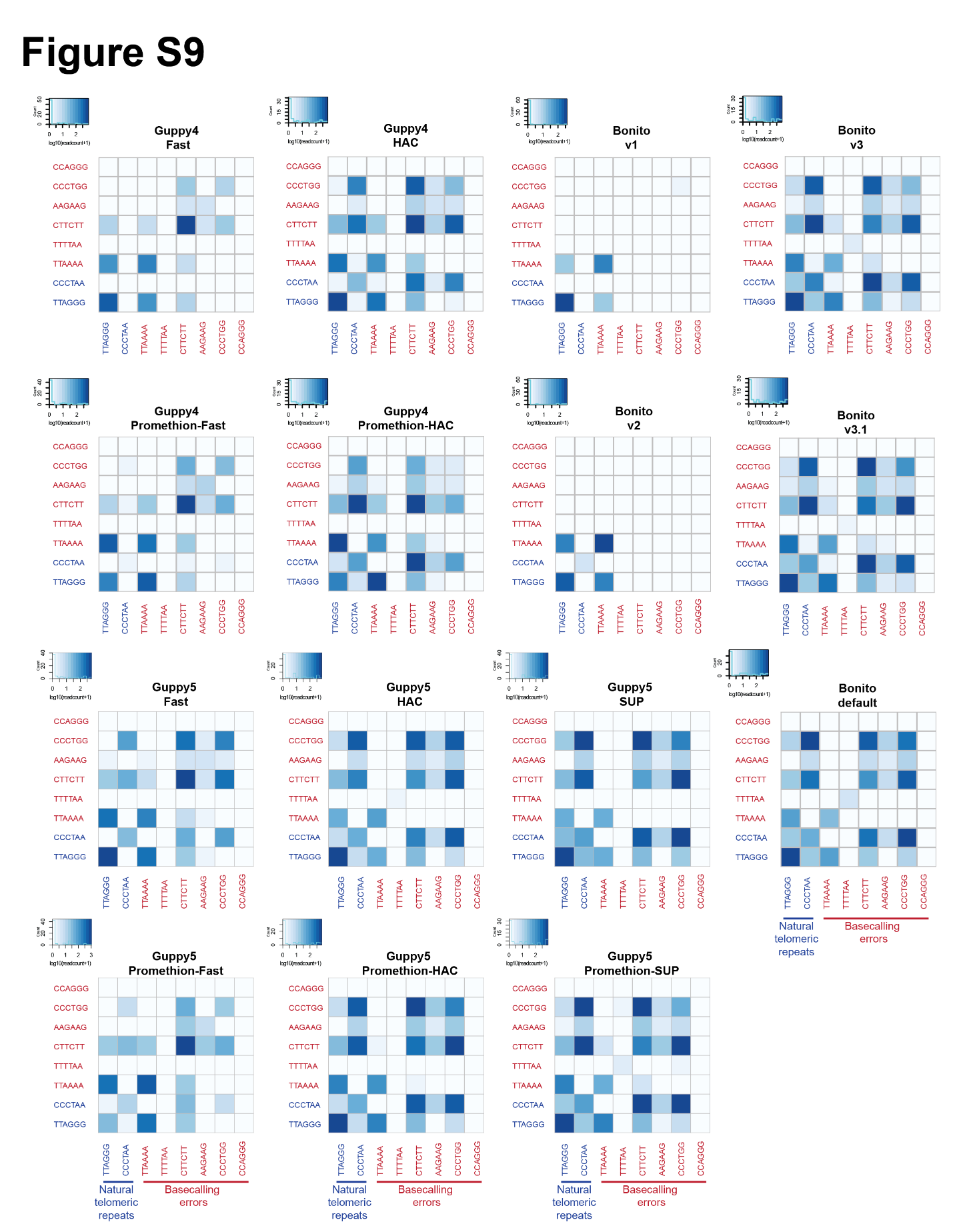
 **Fig. S9 Co-occurrence heatmap for different nanopore basecalling models.** Different nanopore basecallers and basecalling models were applied to the CHM13 nanopore Promethion datasets. The frequency of telomeric repeats and basecalling artefacts observed on reads obtained are as depicted.


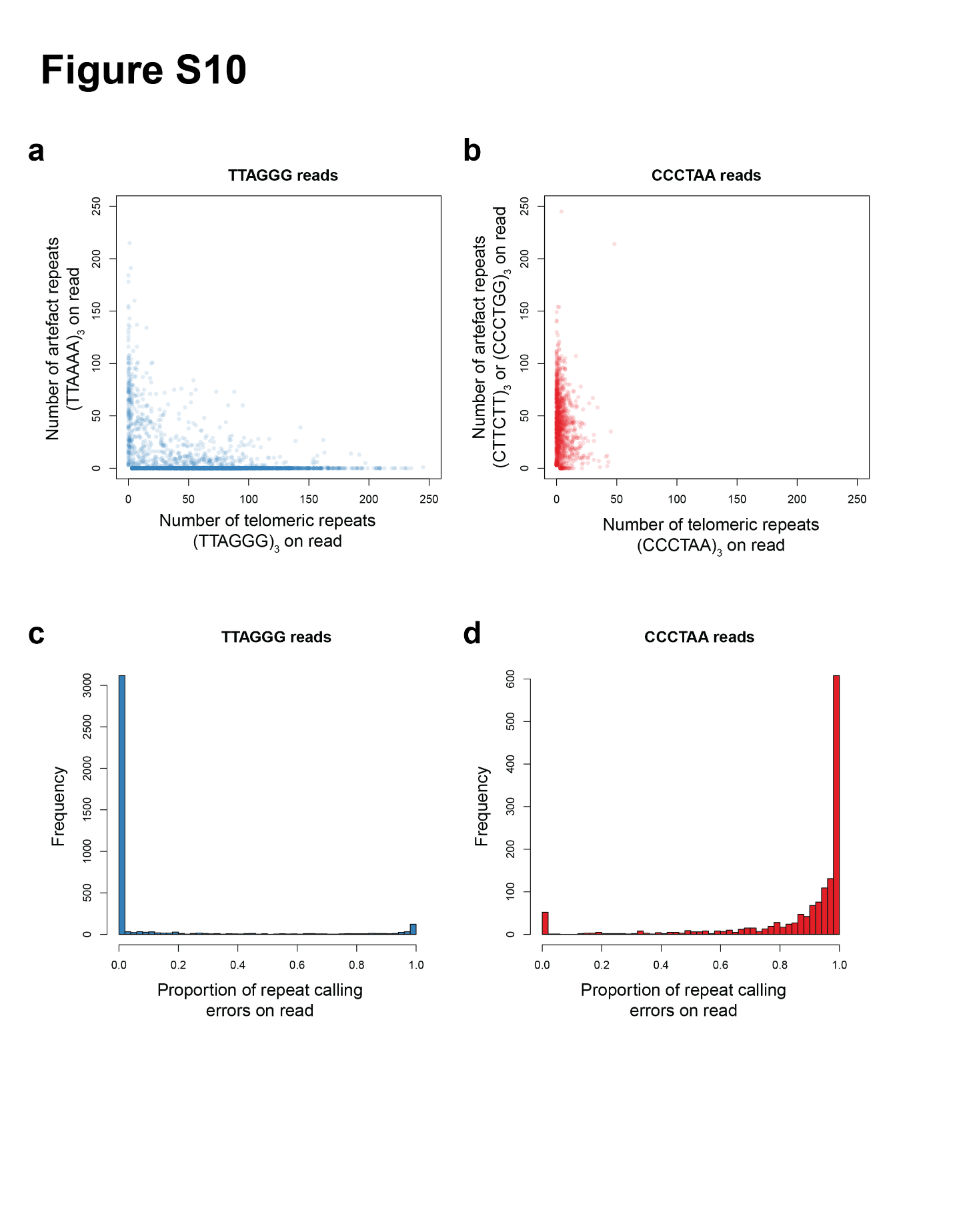


**Fig. S10 Frequency of telomeric repeats and repeat artefacts on each long read. (a-b)** Number of telomeric repeats and artefact repeats on each long-read on the **(a)** “TTAGGG” strand, or the **(b)** “CCCTAA” strand was assessed. Specifically, **(a)** the number of non-overlapping (TTAGGG)_3_ telomeric repeats and (TTAAAA)_3_ artefact repeats on the “TTAGGG” strand were assessed. Also, **(b)** the number of non-overlapping (CCCTAA)_3_ telomeric repeats, and (CTTCTT)_3_ or (CCCTGG)_3_ artefactual repeats were established. The proportion of repeat calling errors on each long read was then assessed for the reads originating from **(c)** the “TTAGGG” strand, or from **(d)** the “CCCTAA” strand are as depicted. Specifically, the proportion of repeat calling errors on each long-read was calculated by dividing the number of artefact repeats on each long-read by the total number of telomeric and artefact repeats.


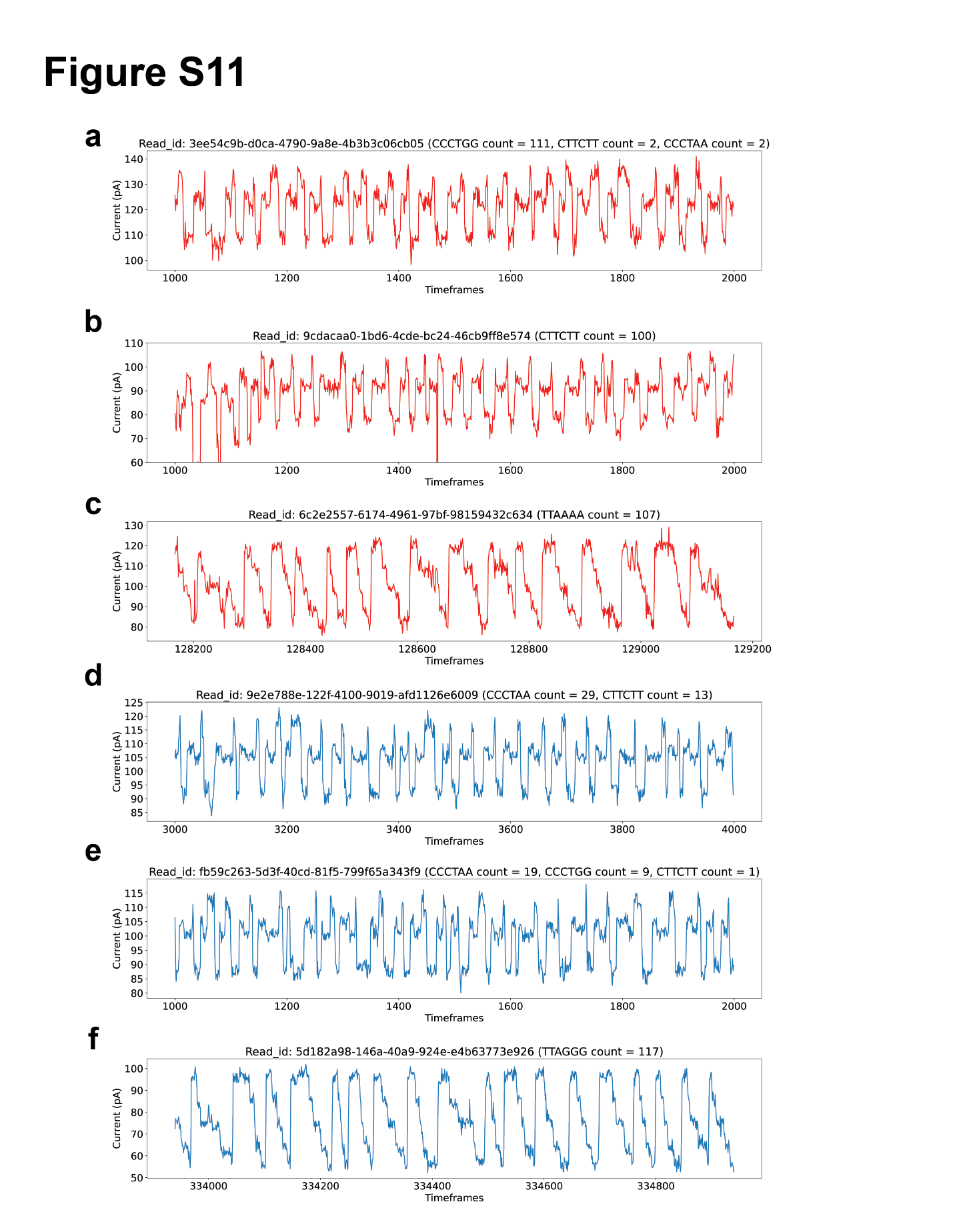
 **Fig. S11 Current profiles for telomeric repeats in reads of low read qualities, or in reads of high read qualities. (a-c)** Telomeric region of three representative reads with which are more error prone (proportion of artefact repeats > 0.95). **(d-f)** Telomeric region of three representative reads which are less error prone (proportion of artefact repeats < 0.4). The corresponding repeat errors observed on each read are as indicated. Plots depict the first 1000^th^-2000^th^ timeframes for reads with (CCCTAA)_n_ repeats or the last 1000^th^-2000^th^ timeframes for reads with (TTAGGG)_n_ repeats, except for (d) where the first 2000 timeframes had a flat current signal. Note that 1000 timeframes correspond to ~105 basepairs given a PromethION read speed of 420 bases/second and a sampling rate of 4000 measurements per second. The number of telomeric repeats ((TTAGGG)_3_ and (CCCTAA)_3_) and repeat artefacts ((TTAAAA)_3_, (CTTCTT)_3_ and (CCCTGG)_3_) on each read is also as indicated in the title of each panel.


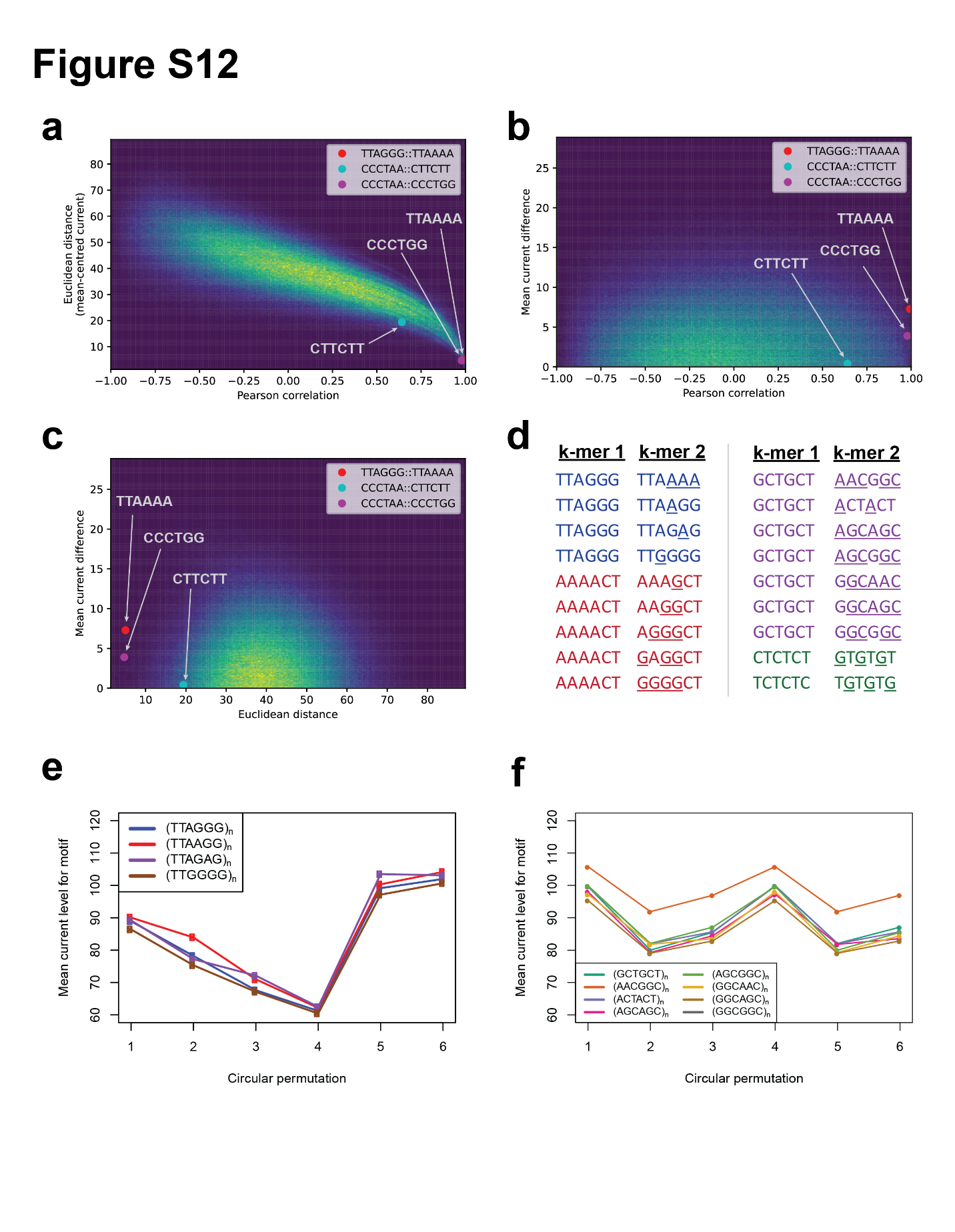
 **Fig. S12 Similarities between current profiles for all possible pairs of 6-mer repeats. (a-c)** Heatmaps depicting the Euclidean distances, Pearson correlation, and mean current differences between current profiles between all possible 6-mer repeat sequences. These are depicted as pairwise plots for **(a)** the Euclidean distances vs. the Pearson correlation, **(b)** the mean current difference vs. the Pearson correlation, and **(c)** the mean current difference vs. the Euclidean distance. The pairwise comparisons between the telomeric repeats and the observed basecalling repeat artifacts are also highlighted in the plots. **(d)** Example pairs of k-mer repeats with similar current profiles are as indicated. The nucleotides in k-mer 2 that differs from k-mer 1 is underlined to highlight the nucleotides that differ between the two types of repeats. **(e-f)** Current profiles for repeats which were predicted to be highly similar to each other. These are depicted for **(e)** TTAGGG telomeric repeats and telomere-like repeat sequences and (**f)** GCTGCT repeat sequences that were highlighted in purple in Additional File 1: Fig. S12d.


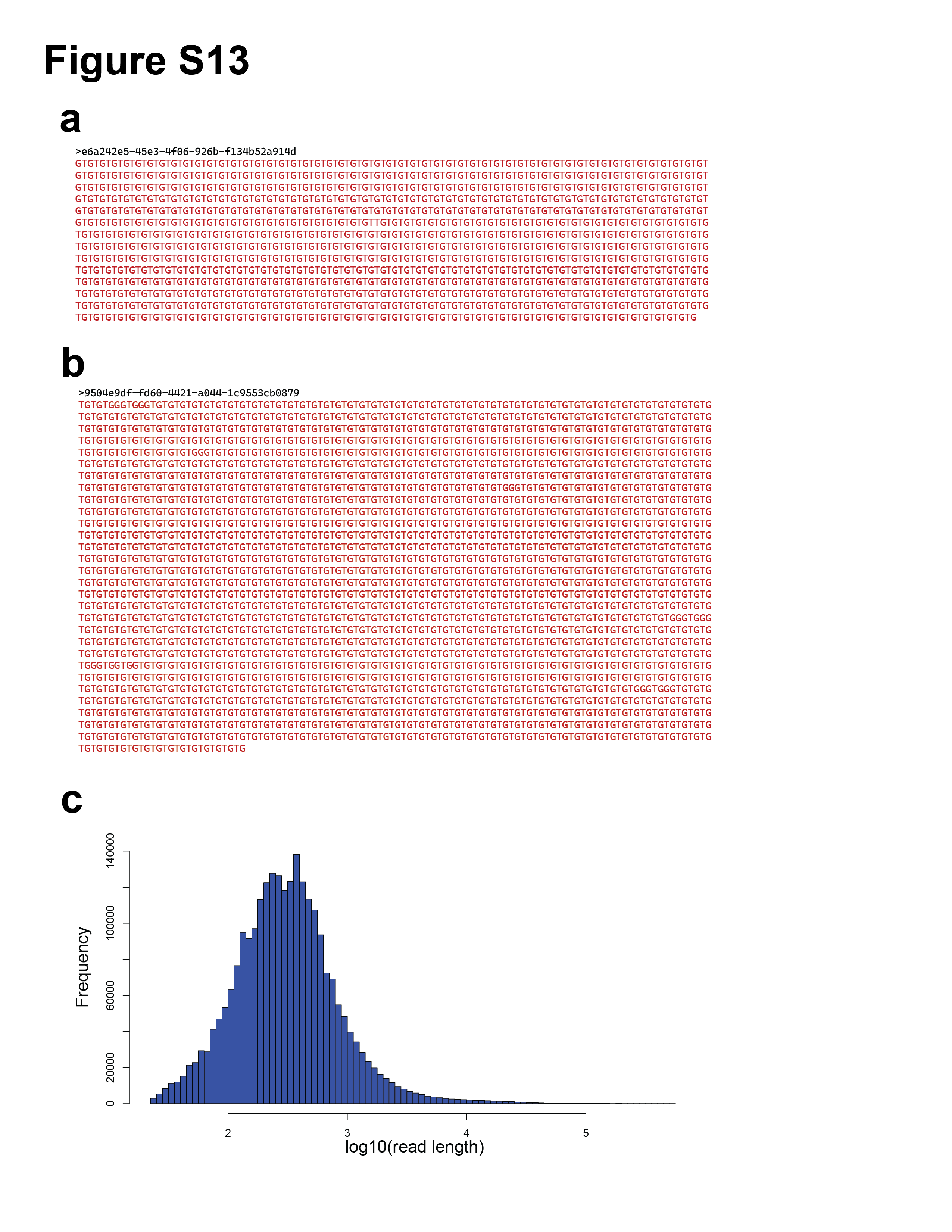
 **Fig. S13 Example of reads with (GT)_n_ repeat sequences in the CHM13 dataset. (a-b)** Two representative reads from the CHM13 nanopore sequencing dataset with (GT)_n_ repeat sequences. **(c)** Read length distribution of unmappable (GT)_n_ repeats (number of repeats ≥12) in the CHM13 nanopore dataset.


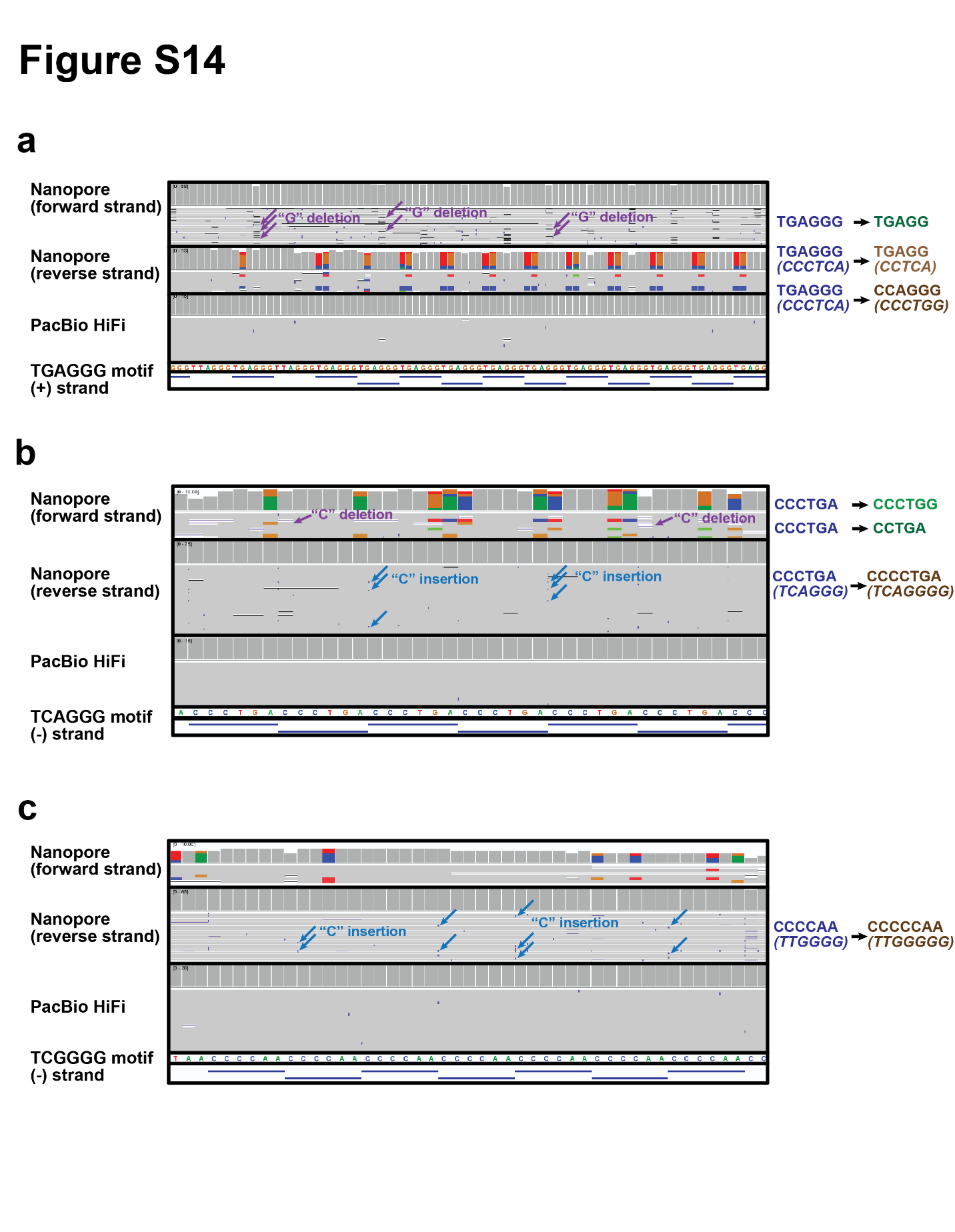
 **Fig. S14 IGV screenshots depicting repeat calling errors observed on telomere-like repeat sequences in the CHM13 dataset. (a)** Repeat calling error observed at the (TGAGGG)_n_ g-type repeat region in the CHM13 sample. The region represented corresponds to chr1:248,385,364-248,385,449. **(b)** Repeat calling error observed at the (TCAGGG)_n_ c-type repeat region in the CHM13 sample. The region represented corresponds to chr1:2,381-2,420. **(c)** Repeat calling error observed at the (TCGGGG)_n_ j-type repeat region in the CHM13 sample. The region represented corresponds to chr1:2,895-2,941.


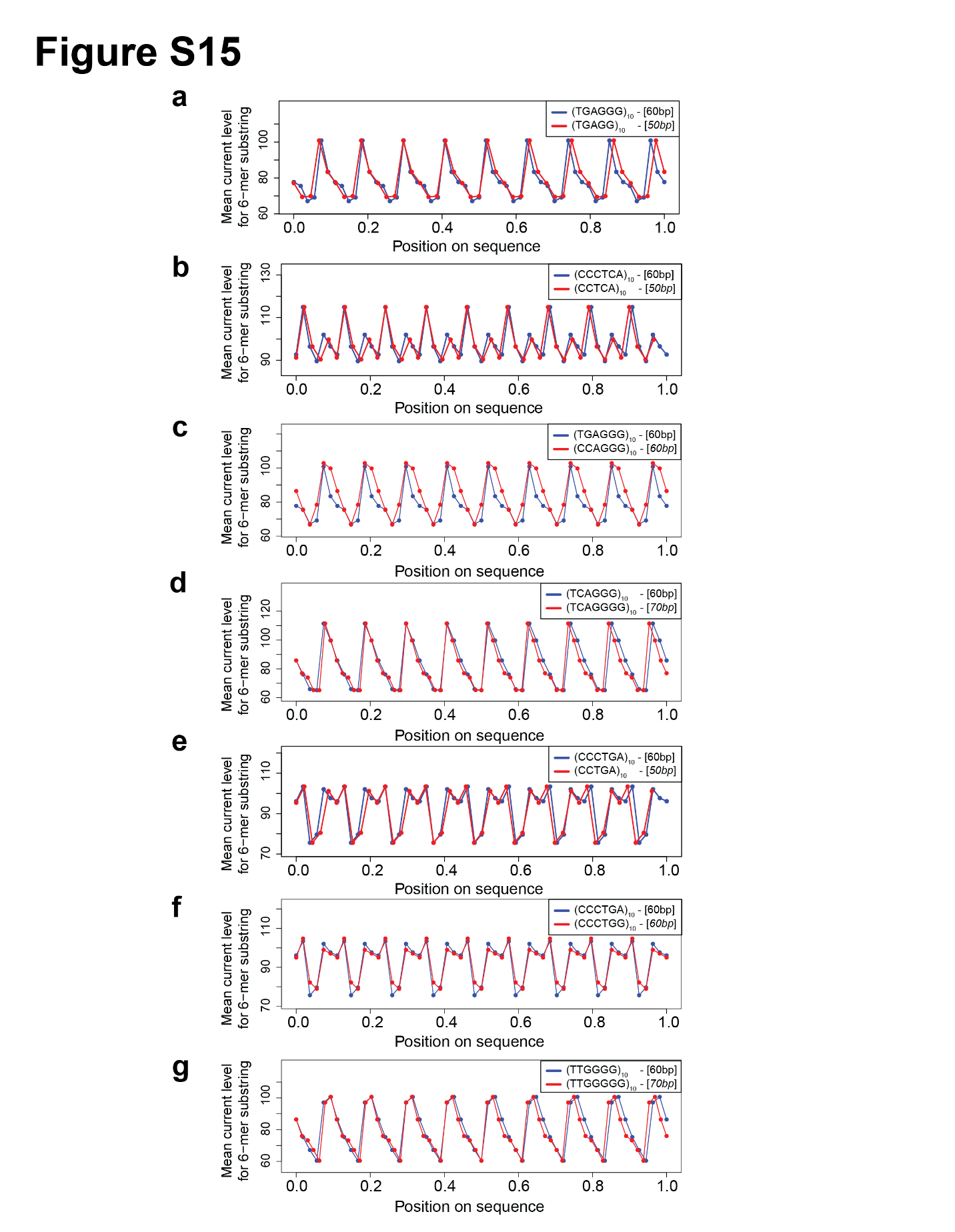
**Fig. S15 Simulated current profiles for 10 consecutive repeats of the telomere-like repeat sequences, and observed repeat calling errors. (a-c)** Simulated current profiles for repeat calling error repeats observed at (TGAGGG)_n_ telomere-like repeat sequences. **(d-f)** Simulated current profiles for repeat calling error repeats observed at (TCAGGG)_n_ telomere-like repeat sequences. **(g)** Simulated current profiles for repeat calling error repeat observed at (TTGGGG)_n_ telomere-like repeat sequences. The three types of telomere-like repeat sequences are colored in blue, while the repeat calling errors are labelled in red. The length of repeat sequences presented in each of the panel are also highlighted in the legend. Note that slight horizontal scaling (<5%) was applied to the red current profiles for panels b and e to allow the current profiles to align better with the blue current profiles. The scaling factor can be read off the plot from the last red dot of each of these plots.


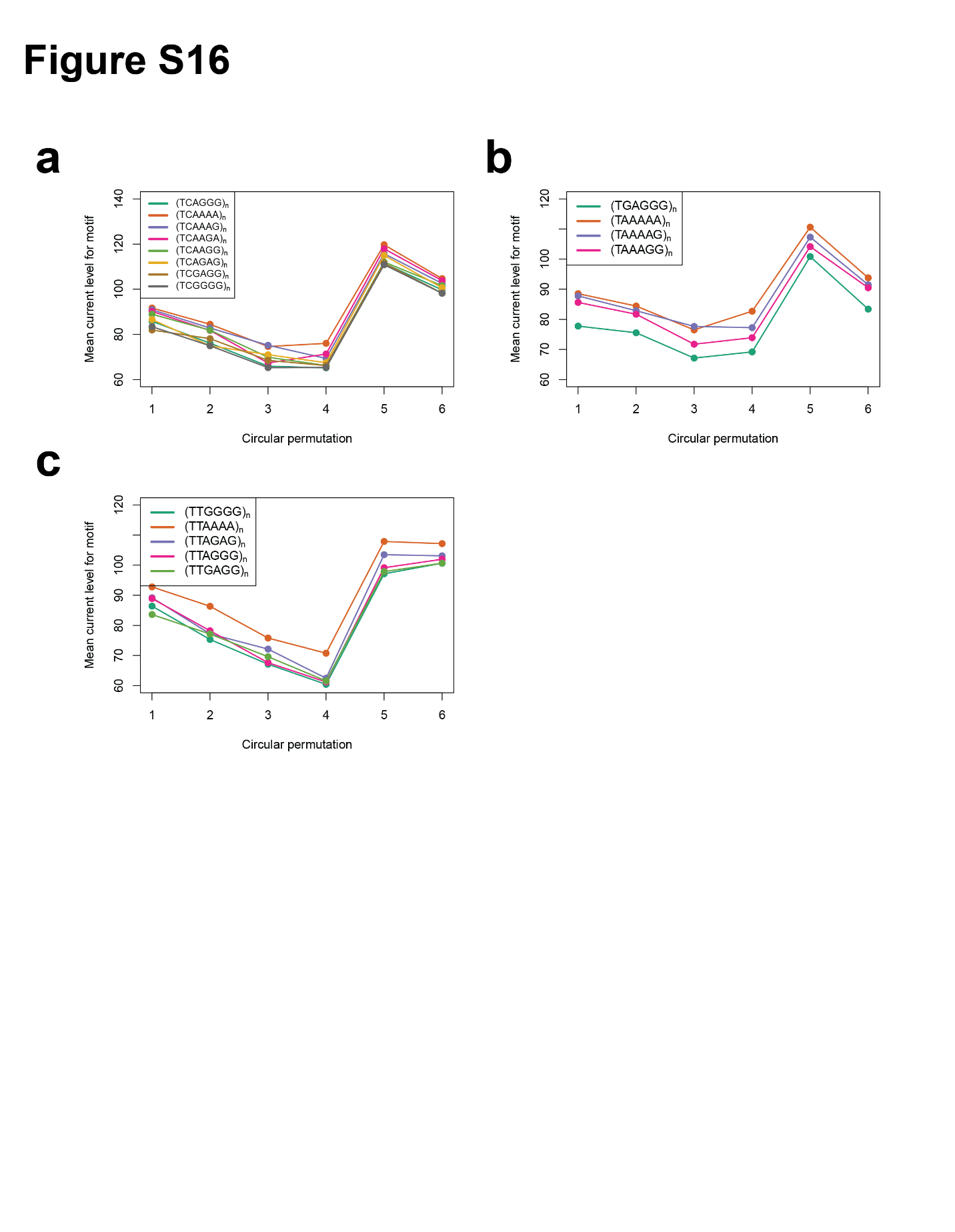
 **Fig. S16 Repeats with predicted similarity in current profiles to the three types of telomere-like repeat sequences.** Repeats that have predicted similarities in current profiles to **(a)** (TCAGGG)_n_, **(b)** (TGAGGG)_n_, and **(c)** (TTGGGG)_n_ are indicated in each of the plots.


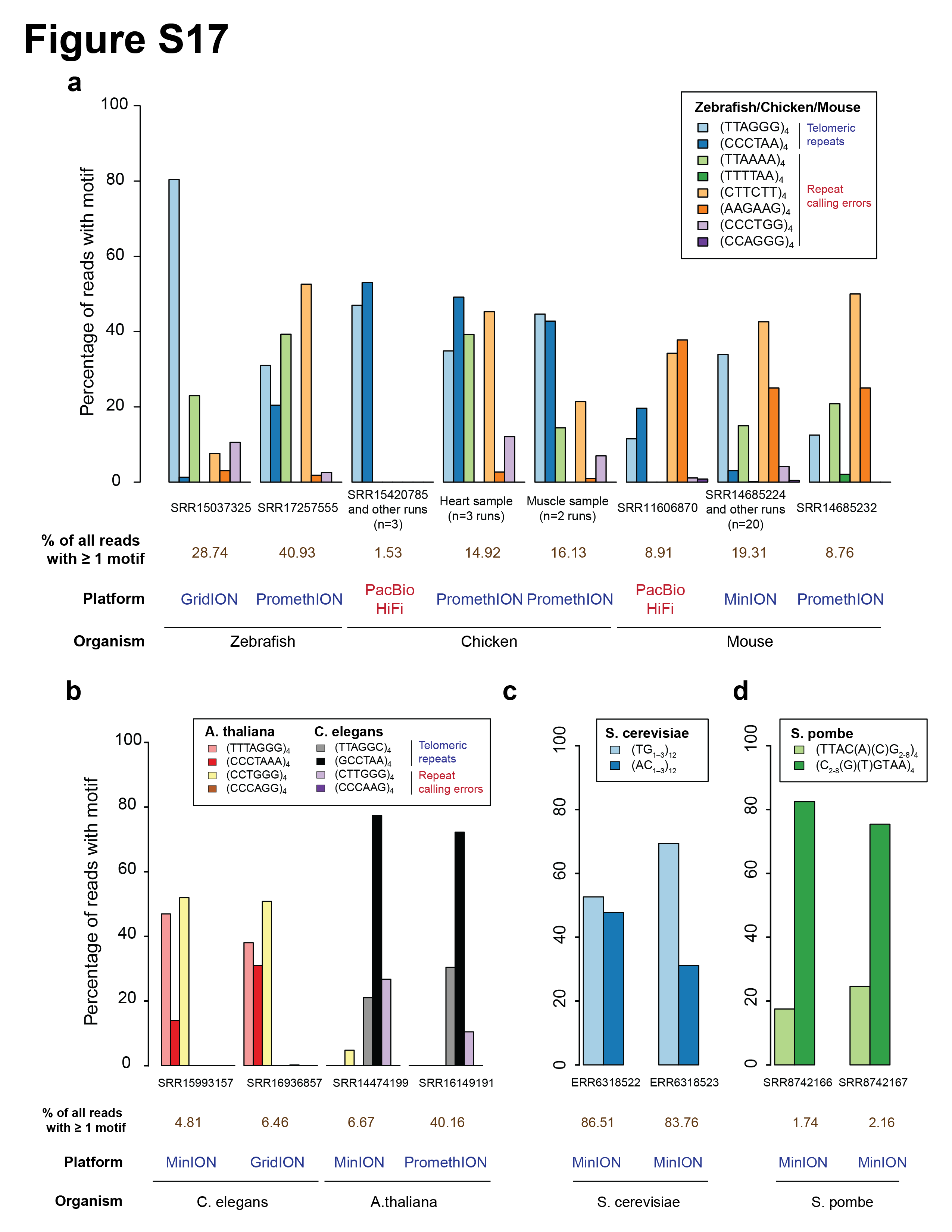
 **Fig. S17 Frequency of natural telomeric repeats, and repeat calling errors in nanopore datasets for each organism assessed.** **(a)** Frequency of natural telomeric repeats and repeat errors at the telomeres in nanopore datasets generated from Zebrafish, Chicken and Mouse. These organisms are known to have (TTAGGG)_n_ repeat sequences at their telomeres. **(b)** Frequency of telomeric repeats and repeat errors in *Arabidopsis thaliana* which is known to have (TTTAGGG)_n_ repeats at its telomeres, and *Caenorhabditis elegans* which is known to have (TTAGGC)_n_ repeats at its telomeres. Frequency of telomeric repeats in two types of yeast, **(c)** *Saccharomyces cerevisiae* which is known to have (TG_1–3_)_n_ repeats at their telomeres, and **(d)** *Schizosaccharomyces pombe* which is known to have TTAC(A)(C)G_2-8_ repeats at the telomeres. Note that the percentages of reads with each motif was normalized to the total count of reads with at least one type repeat sequence in each of the plots. The percentage of reads with at least 1 repeat among all reads analyzed is also as indicated (i.e. at least 1 out of all the repeats analyzed in each panel). This percentage skews to the lower end for some organisms as it was not possible to reliably extract telomeric reads from the terminal ends of the reference genome of all chromosomal arms for all organisms due to the absence of a high-quality reference genome assembly of the sub-telomeres and telomeres.


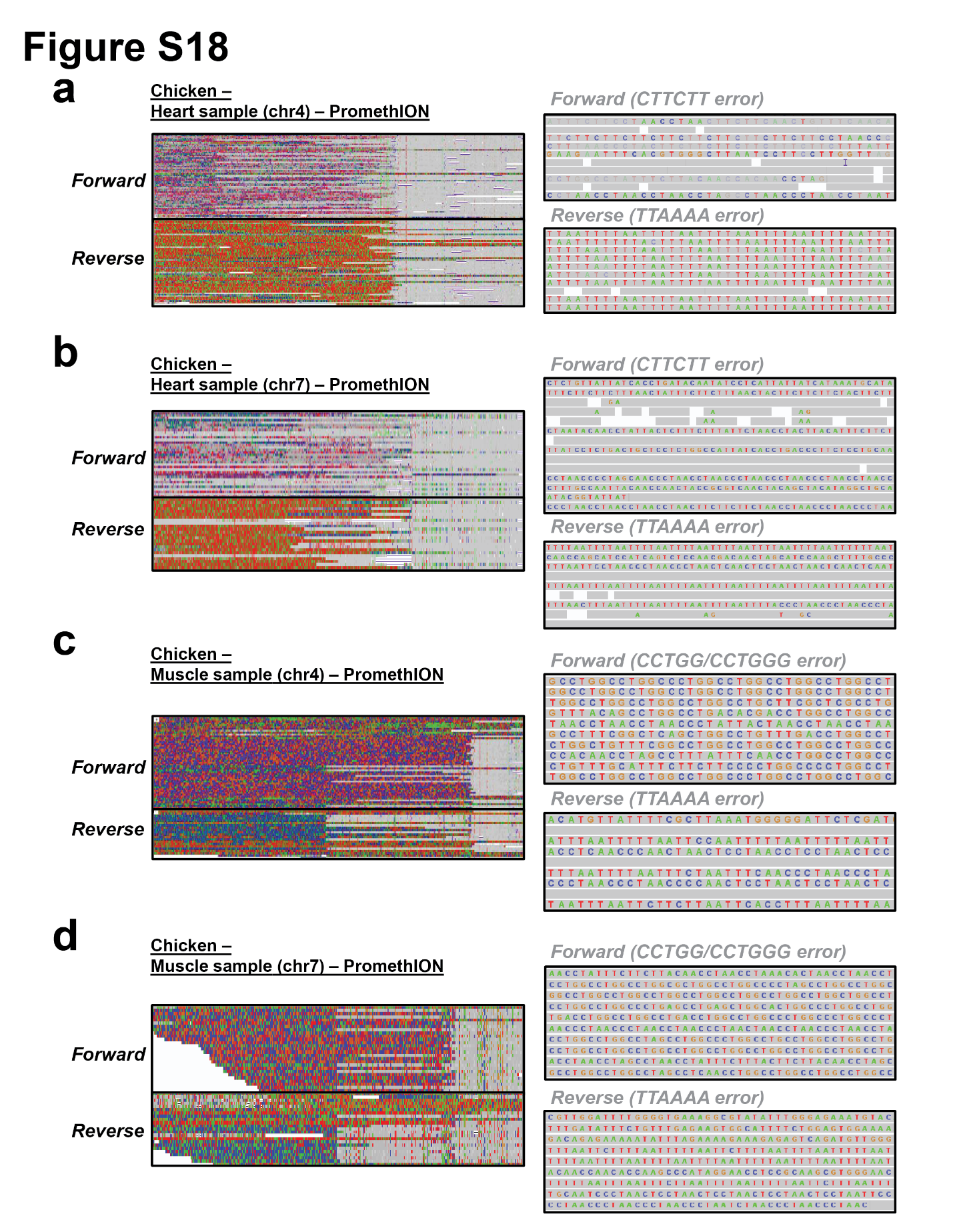
 **Fig. S18 Repeat calling errors are present on the telomeres of Chicken which are characterized by (TTAGGG)_n_ repeat sequences**. Screenshots depicting the forward and reverse strands of two publicly available nanopore datasets corresponding to **(a-b)** a chicken heart sample and **(c-d)** a chicken muscle sample is as depicted. The platforms used for generating these two datasets are also as indicated. Specific zoomed in views of the repeats found on the reads are shown on the right panel. Akin to what we observed at human telomeres that are also characterized by (TTAGGG)_n_ telomeric repeats, we see that (CCCTAA)_n_ repeats were recorded as (CTTCTT)_n_ and (CCTGGG)_n_ repeat errors in the forward strand of the plots. We also see that (TTAGGG)_n_ telomeric repeats are recorded as (TTAAAA)_n_ repeat errors. Specific details of the runs analyzed can be found in **Additional File 2: Table S2**.


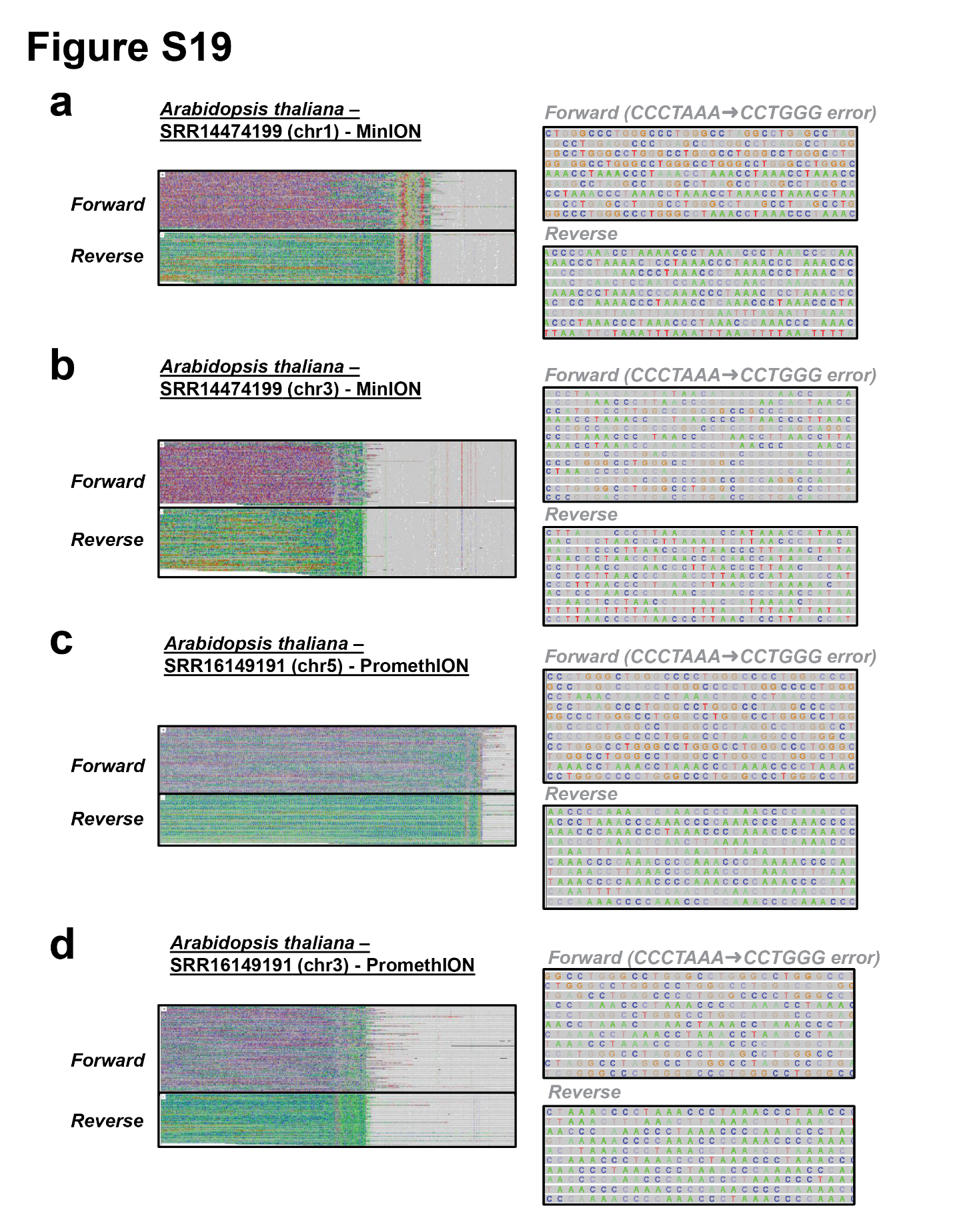
 **Fig. S19 Repeat calling errors are present on the telomeres of *Arabidopsis thaliana* which are characterized by (TTTAGGG)_n_ repeat sequences**. Screenshots depicting the forward and reverse strands of two publicly available nanopore datasets, **(a-b)** SRR14474199 and **(c-d)** SRR16149191 are as depicted. The platforms used for generating these two datasets are also as indicated. Specific zoomed in views of the repeats found on the reads are shown on the right panel. Note that (CCCTAAA)_n_ telomeric repeats were observed as (CCTGGG)_n_ repeat errors in each of the plots.


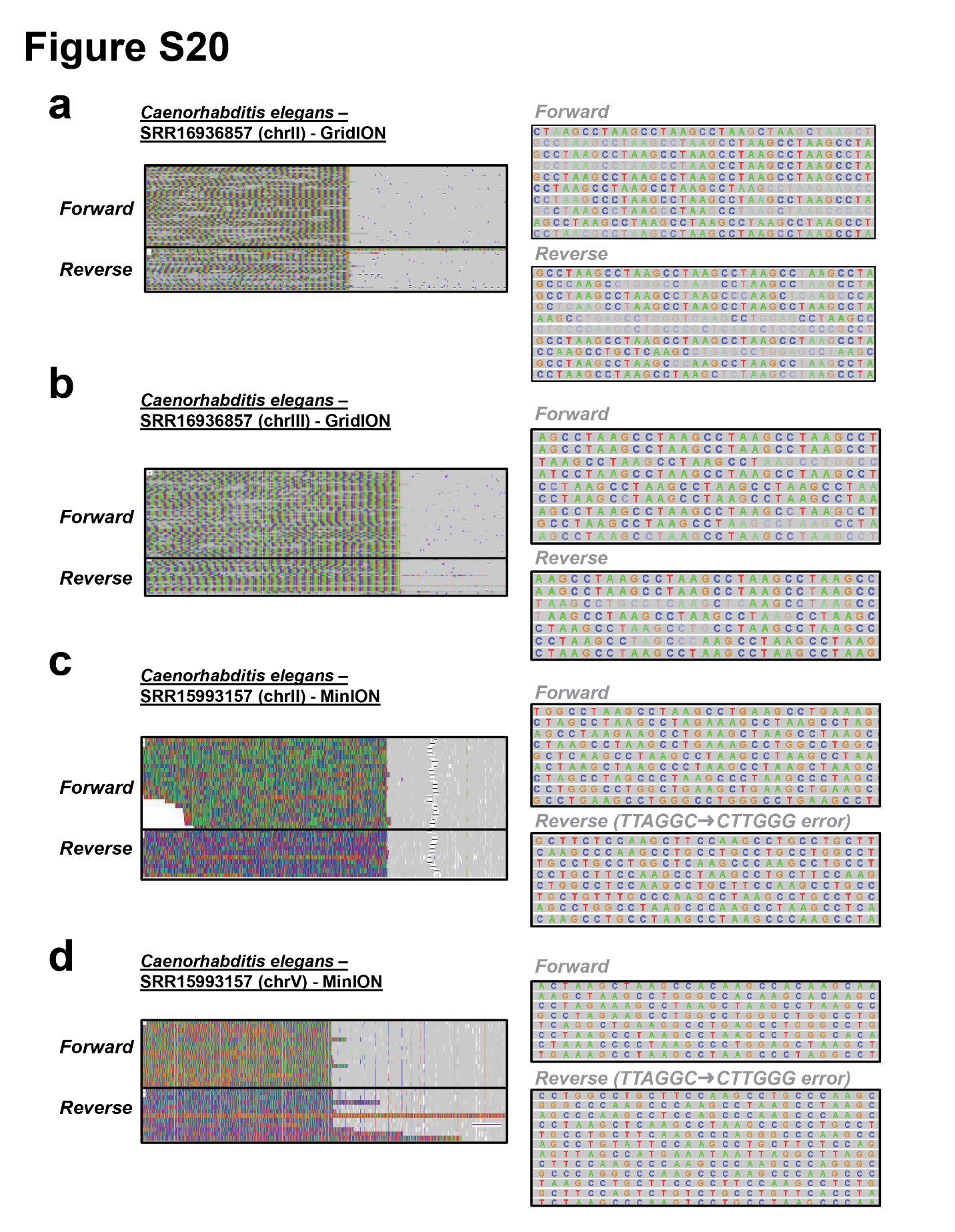
 **Fig. S20 Repeat calling errors are present on the telomeres of some *Caenorhabditis elegans* nanopore datasets. *C. elegans* telomeres are characterized by (TTAGGC)_n_ repeat sequences**. Screenshots depicting the forward and reverse strands of two publicly available nanopore datasets, **(a-b)** SRR16936857 and **(c-d)** SRR15993157 are as depicted. The platforms used for generating these two datasets are also as indicated. Specific zoomed in views of the repeats found on the reads are shown on the right panel. Note that (TTAGGC)_n_ telomeric repeats were observed as (CTTGGG)_n_ repeat errors on the reverse strand of the MinION dataset assessed (SRR15993157), but not in the GridION dataset (SRR16936857).


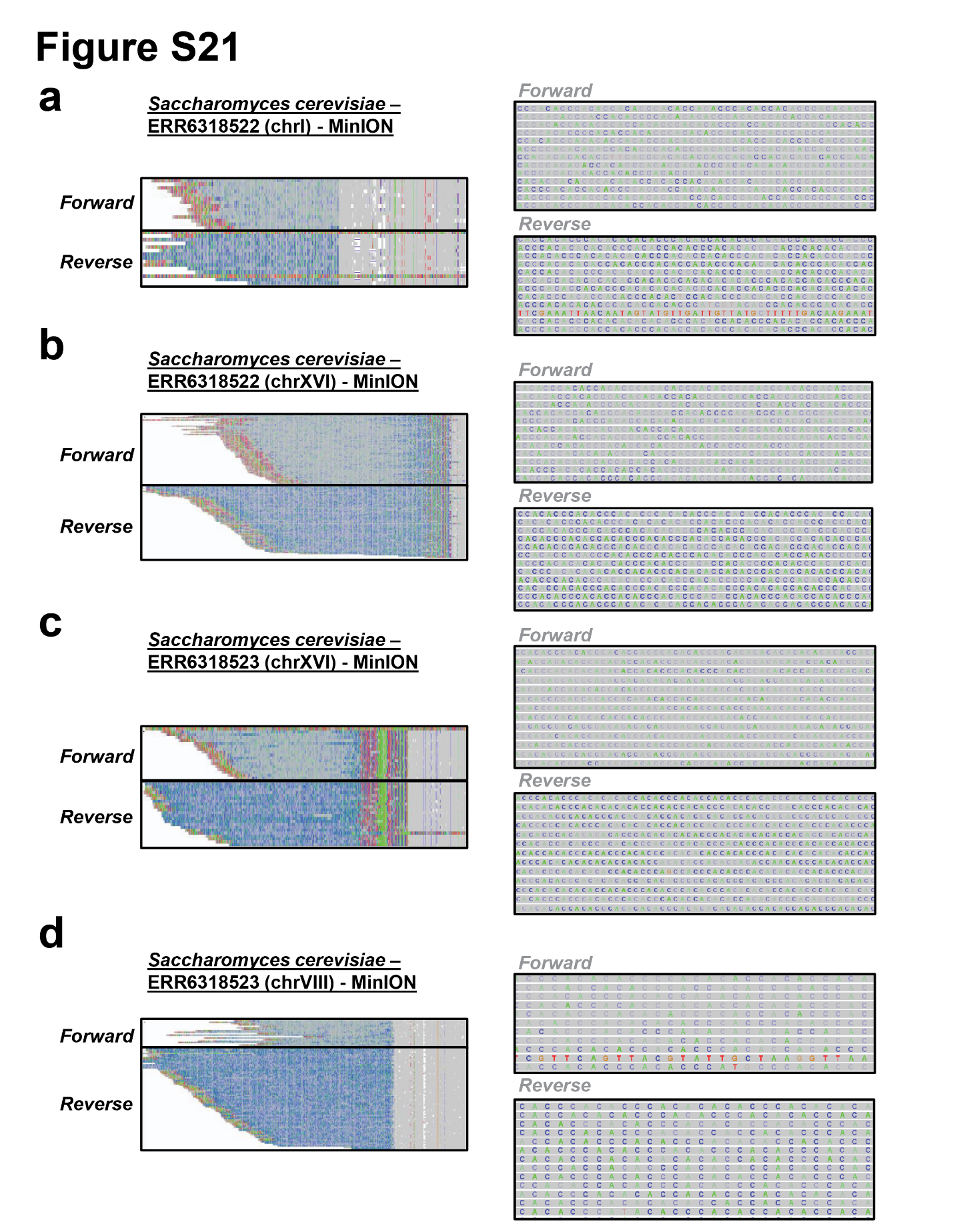
 **Fig. S21 Repeat calling errors are absent on the telomeres of *Saccharomyces cerevisiae* which are characterized by** **(TG_1–3_)_n_ repeat sequences**. Screenshots depicting the forward and reverse strands of two publicly available nanopore datasets **(a-b)** ERR6318522 and **(c-d)** ERR6318523 are as depicted. The platform used for generating these two datasets are also as indicated. Specific zoomed in views of the repeats found on the reads are shown on the right panel. Note that some non-telomeric sequences (indicated by the non-blue sequences) could be observed on the left edge of the reads during IGV visualization. We note that these sequences are concordant with nanopore sequencing adapters which were likely not completely stripped off during basecalling of the reads.


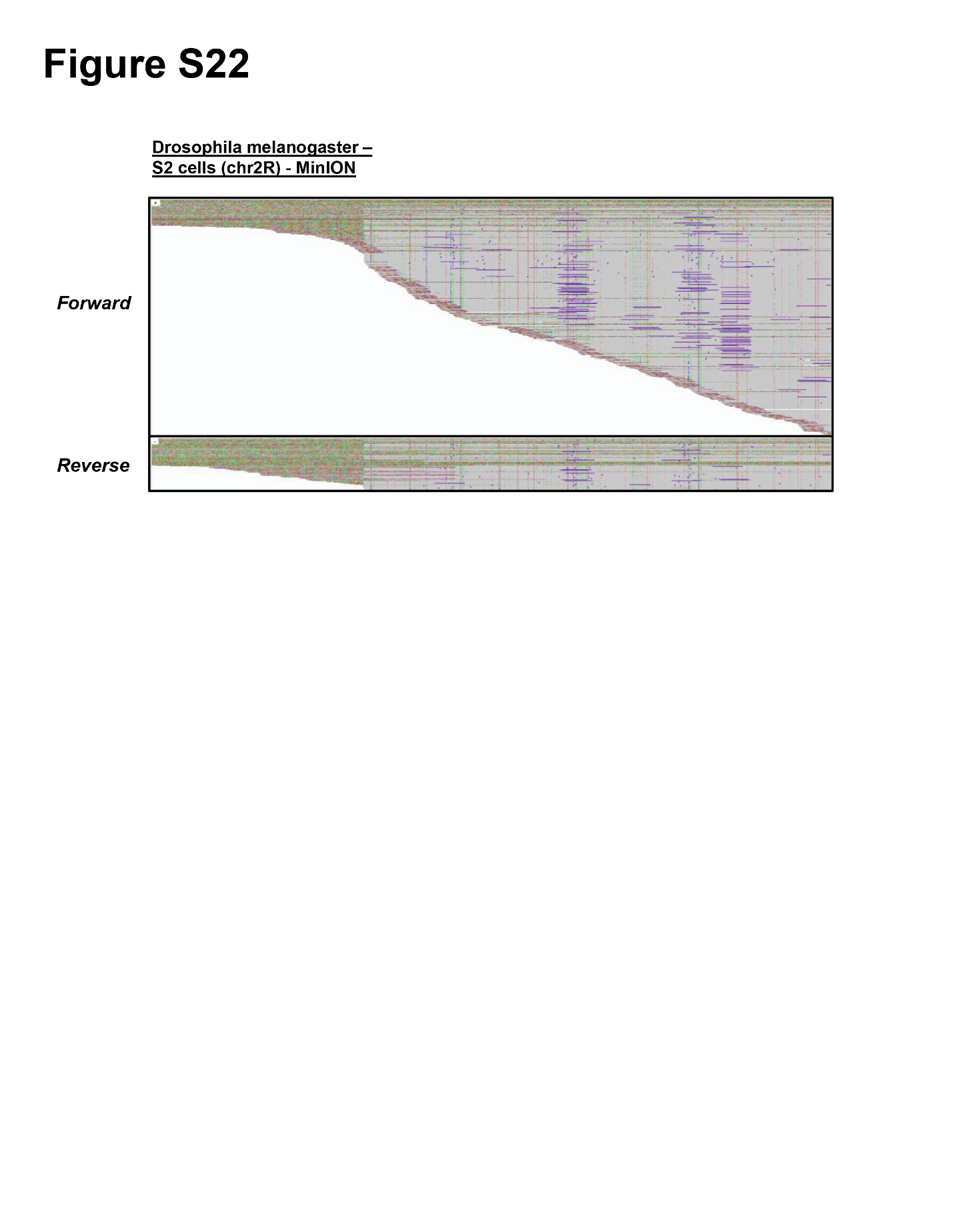


**Fig. S22 No differences in basecalling was observed between different strands at the terminal end of *Drosophila melanogaster*.** Screenshots depicting the forward and reverse strands of a publicly available nanopore datasets is as depicted. Detailed information on the runs used for the analysis can be found in Additional File 2: Table S2.


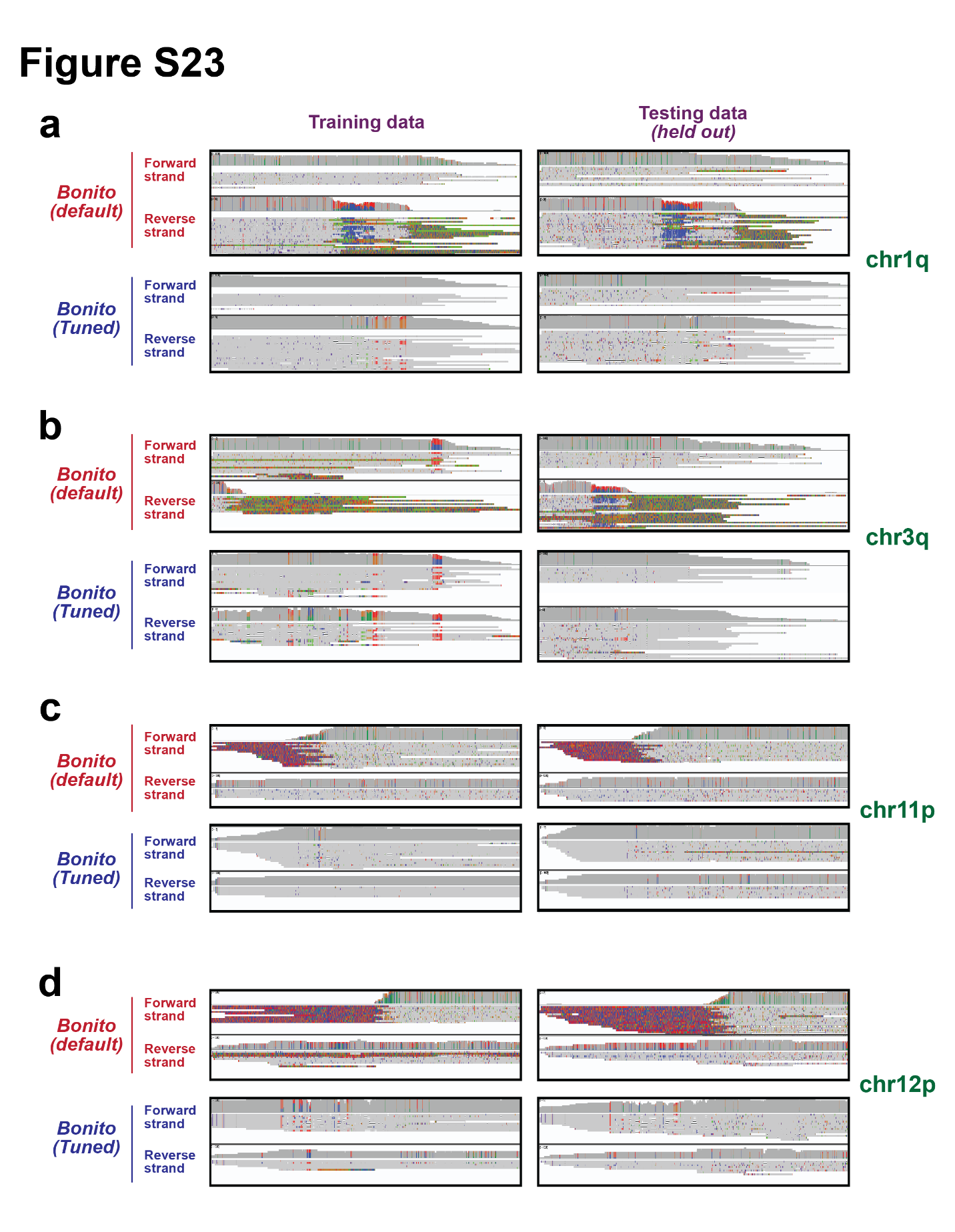
 **Fig. S23 Additional examples for the performance of the tuned bonito basecaller on telomeres on other chromosomal arms.** The tuned model was applied to the training dataset used for model training, and on an additional held out test dataset that was not used during model training. IGV screenshots of the default and tuned bonito basecaller on the training and testing dataset for the chromosomal arms **(a)** chr1q, **(b)** chr3q, **(c)** chr11p and **(d)** chr12p are as depicted. Related to Figure 2b.


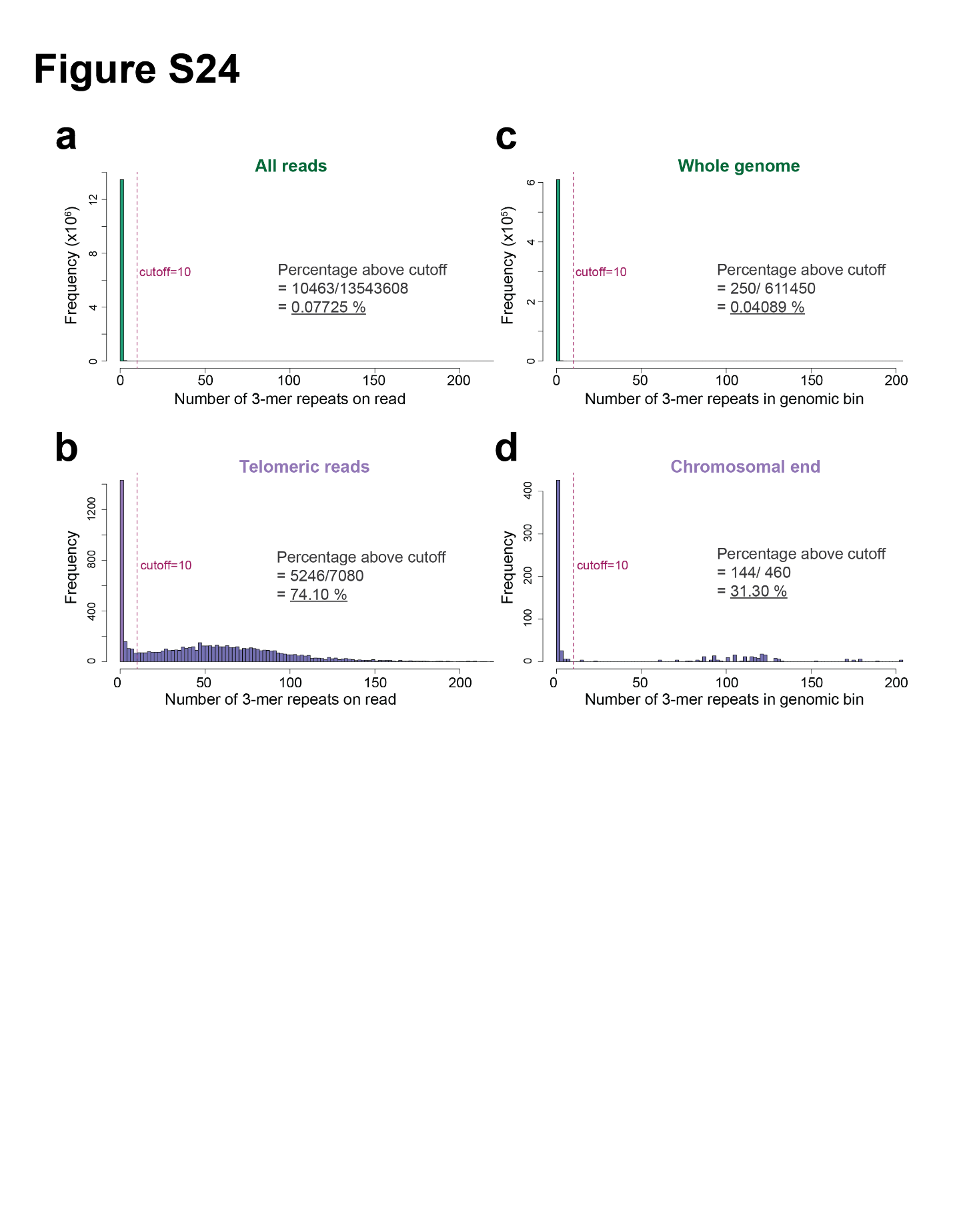
 **Fig. S24 Histograms depicting the frequencies of 3-mer repeats on reads at telomeres and on reads found at the rest of the genome in the CHM13 dataset. (a-b)** The sum of 3-mer telomeric repeats [(TTAGGG)_3_ (CCCTAA)_3_] and basecalling error repeats [(TTAAAA)_3_, (TTTTAA)_3_, (CTTCTT)_3_, (AAGAAG)_3_, (CCCTGG)_3_, (CCAGGG)_3_] on **(a-b)** each long-read or **(c-d)** genomic bin are as depicted on the x-axis of each histogram. The histograms represent the frequency of these repeats on **(a)** all long-reads in the CHM13 dataset, **(b)** telomeric reads in the CHM13 dataset, **(c)** 20 kb genomic bins with 10 kb moving window for the full CHM13 reference genome, **(d)** and for the 10 genomics bins on each chromosomal end of the CHM13 genome.

**Supplementary Tables**

**Table S1 List of k-mers with high similarities in current profiles.** The pearson correlation, Euclidean distance, and mean current difference between each pair of k-mer is as presented in the table.

**Table S2 List of nanopore datasets from different organisms utilized in this study.** The accession number for each of the runs, the nanopore platform used for generating the dataset, and where available the basecaller used for basecalling are as indicated.

**Table S3 Telomeric repeats for organisms assessed and links to publications supporting these telomeric repeats.**
