# Supplementary material for: Identifying and correcting repeat-calling errors in nanopore sequencing of telomeres
Source: Genome Biol. 2022 Aug 26;23:180. doi: 10.1186/s13059-022-02751-6 (PMC9414165; doi:10.1186/s13059-022-02751-6)
Supplement: Supplementary file 3 — Additional file 3: Review history [file 13059_2022_2751_MOESM3_ESM.docx]

Review History

**First round of review**

**Reviewer 1**

**Were you able to assess all statistics in the manuscript, including the appropriateness of statistical tests used?** Yes, and I have assessed the statistics in my report.

**Comments to author:**

===

The authors observed a strand-specific error patterns at telomere repeats in nanopore sequencing data. The authors did extensive analytic experiments and concluded that this was likely caused by basecalling. To correct the errors, the authors fine-tuned the nanopore basecalling models and largely increased the basecalling accuracy of these telomere repeats. Since telomere length/sequences are important for aging and many diseases such as cancers, accurate telomere sequences are fundamental for understanding the underlying mechanisms of aging and many diseases. Technically, the improved long reads are helpful for genomic analysis including de novo assembly and variants characterization. The manuscript is well written and the experiments support the conclusions. I only have several suggestions.

1. The basecalling models induced telomere repeat errors seems a systematic error. Besides human, are there other species showing a similar issue?
2. From the main figures and supplementary figures, it seems that the errors tend to happen on the forward strand of the left arm and the reverse strand of the right arm. Is this true for every chromosome? Are there any differences for the current profiles between the error-prone and less-error reads at either telomere arm? When tuning the bonito model, how are they dealt with? Are they treated similarly or differently?
3. Nanopore has three different sequencing platforms - GromethION, GridION, and MinION. From Figure 1d, different platforms tend to have different preference in terms of basecalling errors. I wonder if this is also considered in the tuning models.
4. The authors did some experiments and suggested that other regions may also have the basecalling errors. This is intriguing and important. A future work might be needed to investigate the genome-wide basecalling errors in repeat regions and provide a solution.
5. The GitHub page should be improved to reflect the dependencies and readability. For example, samtools, minimap2, etc. are needed. And in each folder there is a "main.pl", which is quite confusing when testing.


===

**Reviewer 2**

**Were you able to assess all statistics in the manuscript, including the appropriateness of statistical tests used?** No, I do not feel adequately qualified to assess the statistics.

**Comments to author:**

Tan et al. use the CHM13 genome assembly to identify extensive and specific types of basecalling errors in telomere repeat tracts from nanopore reads derived from this genome. Similar errors occur across nanopore datasets, sequencing platforms, basecallers, and basecalling models; they show that the errors are caused by the high similarity of electrical current profiles between the true telomere repeat sequence motifs (TTAGGG)n and (CCCTAA)n and other specific hexamer repeat motifs during basecalling. The authors also describe a potential remedy for correcting these repeats sequences by fine-tuning the nanopore base-caller using highly accurate telomere and telomere-adjacent sequences from CHM13 (derived from PacBio HiFi sequence) as gold standard to train the basecaller. They demonstrate substantial improvement in accurately calling the telomere motifs in nanopore reads using the trained basecaller.

This is an important advance in understanding the current problems that using nanopore reads may cause in analyzing and distinguishing some types of repeat motifs, and also highlights some current limitations of nanopore basecallers. As such, there will be considerable interest in the nanopore sequencing community; given the critical role of telomeres in biology and human disease and the potential advances in sequencing and assembly that nanopore methods promise for complete telomere to telomere genomes, it should be of interest to a much wider readership as well.

The data and methods presented are fairly solid and support the main conclusions, but there are several questions/issues that should be addressed so the reader can understand the limits of the current telomere correction methods described.

1. The authors identify telomere-containing nanopore reads for further analysis by mapping nanopore reads in the CHM13 dataset back to telomere/subtelomere regions of the CHM13 T2T draft assembly, which included both a HiFi assembly and a nanopore dataset. We need more detail as to how well this nanopore read-mapping actually worked, because traditionally this step has been problematic due to telomere-adjacent segmental duplications that can extend for tens to hundreds of kb from the terminal repeat tract, and single nanopore reads are reported to be somewhat error-prone throughout their read lengths (maybe 93-95 % ??). Was there a size selection to make sure only ultralong reads were used that spanned the segmental duplications ? Were there steps taken to identify and screen out reads that might map to multiple telomeres ? What was the range of nanopore read coverage for individual mapped telomeres ?
2. The centromeric end of the human terminal repeat tract typically contains telomere-like sequences similar but non-identical to the canonical repeats (e.g., (TCAGGG)n, TTGGGG, etc); these are usually telomere-specific and often allele-specific. Does the basecaller perform well enough to detect these amidst the canonical motifs ?

**Authors’ response to reviewers**

Reviewer #1:

The authors observed a strand-specific error patterns at telomere repeats in nanopore sequencing data. The authors did extensive analytic experiments and concluded that this was likely caused by basecalling. To correct the errors, the authors fine-tuned the nanopore basecalling models and largely increased the basecalling accuracy of these telomere repeats. Since telomere length/sequences are important for aging and many diseases such as cancers, accurate telomere sequences are fundamental for understanding the underlying mechanisms of aging and many diseases. Technically, the improved long reads are helpful for genomic analysis including de novo assembly and variants characterization. The manuscript is well written and the experiments support the conclusions. I only have several suggestions.

We are happy to note that the reviewer thinks our manuscript is well written and that the experiments we performed support the conclusions.

1. The basecalling models induced telomere repeat errors seems a systematic error. Besides human, are there other species showing a similar issue?

The reviewer raised an intriguing and important question with regards to whether telomere repeat calling errors are also observed in other species besides human. To address this, we obtained Nanopore genome sequencing dataset corresponding to eight well-studied model organisms covering a wide spectrum of the tree of life from the NCBI SRA database **(Revised Figure 1g; Reproduced as Response Table 1)**. These eight different organisms are *Arabidopsis thaliana*, *Caenorhabditis elegans*, *Gallus gallus* (chicken), *Drosophila melanogaster*, *Mus musculus* (mouse), *Saccharomyces cerevisiae*, *Schizosaccharomyces pombe*, and *Danio rerio* (zebrafish). We had decided to focus our analysis on these model organisms as these organisms are more extensively studied, and there is probably greater interest in whether repeat calling errors occur on the telomeres of these organisms. These model organisms were also chosen in part due to the presumed availability of a high-quality reference genome assembly which makes it easier to assess the presence of the repeat calling errors.

**Response Table 1 Summary of organisms analyzed, the type of telomere repeats that are known to occur on these organisms, and if repeat calling errors were observed on telomeres in these organisms.**

As raw signal data was unavailable for these datasets, we had decided to analyze the basecalled sequences published by the authors on the SRA database. We note that this might introduce some degree of heterogeneity in our results since the basecaller and basecalling model used for each dataset may be different. Nonetheless, without the raw signal data made available to us, it is not possible for us to perform a more rigorous analysis of these samples. That said, we believe that the analysis would still give us a rough sense of the types of errors that might be observed in each organism using the Nanopore platform.

With that, we mapped and analyzed Nanopore obtained from eight different organisms and observed repeat calling errors at five out of these eight organisms analyzed **(Revised Figure 1g and Supplementary Figure 17; Reproduced as Response Table 1 and Response Figure 1)**. Specifically, we observed similar telomeric repeat errors as in humans in *A. thaliana* which are known to have 7-mer (TTTAGGG)_n_ repeats (note that humans have a slightly different repeat sequence of TTAGGG) **(Revised Supplementary Figure 17b and 19; Reproduced as Response Figure 1b and 2)**, which suggests that these repeats need not be 6-mer repeats (approximated number of nucleotides detected by the nanopore at each time) to be observed. At the same time, we did not observe these repeat errors for *S. cerevisiae* which is known to have (TG_1–3_)_n_ repeats at their telomeres **(Revised Supplementary Figure 21)**, suggesting that these repeat errors do not occur on telomeres of all organisms. As expected, we also observed repeat calling errors on telomeres in organisms with (TTAGGG)_n_ type repeats **(Revised Supplementary Figure 17a and 18)**, akin to what we observed in humans.

Together, our results suggest that repeat calling errors in Nanopore sequencing can be observed at telomeres of other organisms beyond just human telomeres.

**Response Figure 1 Frequency of natural telomeric repeats and repeat calling errors in Nanopore datasets for each organism assessed.** **(a)** Frequency of natural telomeric repeats and repeat errors at the telomeres in Nanopore datasets generated from Zebrafish, Chicken and Mouse. These organisms are known to have (TTAGGG)_n_ repeat sequences at their telomeres. Frequency of telomeric repeats in two types of yeast, **(b)** *Saccharomyces cerevisiae* which is known to have (TG_1–3_)_n_ repeats at their telomeres, and **(c)** *Schizosaccharomyces pombe* which is known to have TTAC(A)(C)G_2-8_ repeats at the telomeres. **(d)** Frequency of telomeric repeats and repeat errors in *Arabidopsis thaliana* which is known to have (TTTAGGG)_n_ repeats at its telomeres, and *Caenorhabditis elegans* which is known to have (TTAGGC)_n_ repeats at its telomeres. Note that the percentages of reads with each motif was normalized to the total count of reads with at least one type repeat sequence in each of the plots. The percentage of reads with at least 1 repeat among all reads analyzed is also as indicated (i.e. 1 out of the 14 types of repeats analyzed in panel a). This percentage skews to the lower end for some organisms as it was not possible to reliably extract telomeric reads by going to the terminal ends of the reference genome of all chromosomal arms for all organisms due to the absence of a high quality reference genome assembly of the sub-telomeres and telomeres.

**Response Figure 2 Repeat calling errors are present on the telomeres of *Arabidopsis thaliana* which are characterized by (TTTAGGG)_n_ repeat sequences**. Screenshots depicting the forward and reverse strands of two publicly available nanopore datasets, **(a-b)** SRR14474199 and **(c-d)** SRR16149191 are as depicted. The platforms used for generating these two datasets are also as indicated. Specific zoomed in views of the repeats found on the reads are shown on the right panel. Note that (CCCTAAA)_n_ telomeric repeats were observed as (CCTGGG)_n_ repeat errors in each of the plots.

2. From the main figures and supplementary figures, it seems that the errors tend to happen on the forward strand of the left arm (CCCTAA) and the reverse strand of the right arm (CCCTAA). Is this true for every chromosome? Are there any differences for the current profiles between the error-prone and less-error reads at either telomere arm? When tuning the bonito model, how are they dealt with? Are they treated similarly or differently?

First, the reviewer raised the question as to whether the repeat calling errors tend to happen on the forward strand of the left arm (CCCTAA) and the reverse strand of the right arm (CCCTAA) for every chromosome. To address this, we analyzed the frequency of telomeric repeats and repeat calling errors on each chromosomal arm in the CHM13 dataset **(Revised Supplementary Figure 4; Reproduced as Response Figure 3)**. We see that these repeat calling errors can be observed on all chromosomal arms in the CHM13 dataset. Of note, we see that the percentage of reads with each type of errors on the (CCCTAA)_n_ strand (i.e. (CTTCTT)_n_ and (CCCTGG)_n_ errors) are more or less similar (~15-20%) across all the chromosomal arms (except for chr3p and chr4p where the percentage of reads with CTTCTT repeats was much elevated to ~50%) **(Revised Supplementary Figure 4; Reproduced as Response Figure 3)**. Similarly, we also see that the percentage of reads with errors on the (TTAGGG)_n_ strand (i.e. (TTAAAA)_n_ errors) is also rather similar (~15%) across the different chromosomal arms (except for chr14p) **(Revised Supplementary Figure 4; Reproduced as Response Figure 3)**. Further, we see that the proportion of reads with (CCCTAA)_n_ telomeric repeats are always less than the proportion of reads with (TTAGGG)_n_ telomeric repeats across all chromosomal arms. Noting the results above, we think that the errors on each of the chromosomal arms are likely to be similar to each other, with the majority of errors on the (CCCTAA)_n_ strand rather than the (TTAGGG)_n_ strand.

Second, the reviewer also raised another interesting question as to whether we see any differences in the current profiles between the error-prone and the less error prone reads. To address this, we first established which reads are more error-prone or less error-prone. To do so, we calculated the number of telomeric repeats ((TTAGGG)_3_ and (CCCTAA)_3_), and artefact repeats ((TTAAAA)_3_, (CCCTGG)_3_, (CTTCTT)_3_) on each long-read **(Revised Supplementary Figure 10a-b)**. The proportion of repeat-calling errors on each read can then be established by the dividing the number of artefact repeats by the total number of telomeric and artefact repeats **(Revised Supplementary Figure 10c-d)**. While the majority of long-reads (69.5%) on the “CCCTAA” strand had an error proportion of >90%, only 5.2% of long-reads on the “TTAGGG” strand had an error proportion of <10%, suggesting that that the repeat calling errors occur more frequently on the “CCCTAA” strand than on the “TTAGGG” strand. We then examined the current profiles of the more error-prone reads (i.e. reads with a higher proportion of repeat calling errors) and the less error-prone reads. Of note, we were not able to observe a significant visual difference in current profiles between the reads with a higher proportion (>0.9) of repeat calling errors **(Revised Supplementary Figure 11a-c; Reproduced as Response Figure 4a-c)** versus the reads with a lower proportion (<0.4) of repeat calling errors **(Revised Supplementary Figure 11d-f; Reproduced as Response Figure 4d-f)**. Together, our results suggest that while there was different propensity for error repeats to be observed on each long-read, we were unable to observe a significant visual difference in current profiles between these reads.

Third, the reviewer also asked a question about whether we treated the error-prone vs. less error-prone reads differently during model training. When tuning the bonito model, we did not deal with this explicitly (i.e. by splitting the reads into more error-prone vs. less error-prone reads). However, assuming that there are some systematic features associated with the error prone vs. less error-prone reads, these features could potentially be captured during training of the deep learning model. As such, there may not be an explicit need to deal with these two classes of reads separately. As such, we have treated all reads (both error-prone and less error-prone reads) similarly during processing, though we like to note that it is possible that the deep learning model used for basecalling might have been able to capture relevant features to assess these reads differently.

**Response Figure 3 Frequency of telomeric repeats and repeat artefacts on each chromosomal arm.** Frequency of telomeric repeats, and repeat errors on each chromosomal arm in the CHM13 dataset as depicted. Nanopore reads presented in this plot were basecalled using the Bonito basecaller.

 **Response Figure 4 Current profiles for telomeric repeats in reads of low read qualities, or in reads of high read qualities. (a-c)** Telomeric region of three representative reads with which are more error prone (proportion of artefact repeats > 0.95). **(d-f)** Telomeric region of three representative reads which are less error prone (proportion of artefact repeats < 0.4). The corresponding repeat errors observed on each read are as indicated. Plots depict the first 1000^th^-2000^th^ timeframes for reads with (CCCTAA)_n_ repeats or the last 1000^th^-2000^th^ timeframes for reads with (TTAGGG)_n_ repeats, except for (d) where the first 2000 timeframes had a flat current signal. Note that 1000 timeframes correspond to ~105 basepairs given a PromethION read speed of 420 bases/second and a sampling rate of 4000 measurements per second. The number of telomeric repeats ((TTAGGG)_3_ and (CCCTAA)_3_) and repeat artefacts ((TTAAAA)_3_, (CTTCTT)_3_ and (CCCTGG)_3_) on each read is also as indicated in the title of each panel.

3. Nanopore has three different sequencing platforms - GromethION, GridION, and MinION. From Figure 1d, different platforms tend to have different preference in terms of basecalling errors. I wonder if this is also considered in the tuning models.

While we note that the Guppy basecalling software has different basecalling models for the PromethION vs. MinION/GridION platforms, we did not explicitly train separate models for these three sequencing platforms. A key reason we had decided against doing this is because the Bonito basecalling model that our work was based upon on, was designed to be applied to datasets generated by all three nanopore sequencing platforms. Even so, the Bonito basecaller provided by Oxford Nanopore was still able to achieve high level of sequencing accuracy at non-repetitive regions of the genome for each of these three platforms. As such, even though we agree with the reviewer that developing a platform-specific basecalling model for the two main types of Nanopores (GridION/MinION vs. PromethION) can potentially help further improve the accuracy of basecalls at telomeres, we think the improvements are likely to be marginal.

4. The authors did some experiments and suggested that other regions may also have the basecalling errors. This is intriguing and important. A future work might be needed to investigate the genome-wide basecalling errors in repeat regions and provide a solution.

The reviewer raised an intriguing question about the genome-wide basecalling errors in repeat regions. As the reviewer highlighted, this is beyond the scope of our current work, and we have modified our main text to highlight this as a potential future work. Specifically, we have added the statement “***An extensive evaluation of genome-wide basecalling errors in repeat regions is also needed in the future given our observations at telomeric regions****.”* to the concluding paragraph of our revised manuscript as the reviewer has suggested.

That said, we have also looked into telomere-like repeat sequences (i.e (TGAGGG)_n_, (TCAGGG)_n_ and (TCGGGG)_n_ repeats) as Reviewer 2 has suggested. We see that telomere-repeat calling errors are also observed in these three types of repeat sequences to some degree **(Revised Supplementary Figure 14)**. Interestingly, we see that the length of these repeat calling errors need not be of the same length and can also occur between 6-mer repeats on these telomere-like sequences and 5-mer error repeat sequences like for (TGAGGG)_n_ vs. (TGAGG)_n_ **(Revised Supplementary Figure 14a)**. These errors can also occur between the 6-mer telomere-like repeat sequence and the 7-mer repeat calling errors like (TCAGGG)_n_ vs. (TCAGGGG)_n_. Interestingly, a further investigation of the simulated current profiles of these repeat sequences of different lengths showed that they have similar current profiles despite having different sequence lengths **(Revised Supplementary Figure 15)**. Together, this suggests that these repeat calling errors can occur at other repeat regions beyond the telomeres.

5. The GitHub page should be improved to reflect the dependencies and readability. For example, samtools, minimap2, etc. are needed. And in each folder there is a "main.pl", which is quite confusing when testing.

We like to thank the reviewer for the suggestion and recommendations to improve the dependencies and reliability of our GitHub page. As the reviewer suggested, we have updated our GitHub repository with information on the software dependencies. Further, we have also created a script named “fullpipeline.pl” that allows the user to apply the basecalling model we generated in a single command. We believe this would make the basecalling model we generated easier to apply on telomeric reads in future datasets.

Reviewer #2:

Tan et al. use the CHM13 genome assembly to identify extensive and specific types of basecalling errors in telomere repeat tracts from nanopore reads derived from this genome. Similar errors occur across nanopore datasets, sequencing platforms, basecallers, and basecalling models; they show that the errors are caused by the high similarity of electrical current profiles between the true telomere repeat sequence motifs (TTAGGG)n and (CCCTAA)n and other specific hexamer repeat motifs during basecalling. The authors also describe a potential remedy for correcting these repeats sequences by fine-tuning the nanopore base-caller using highly accurate telomere and telomere-adjacent sequences from CHM13 (derived from PacBio HiFi sequence) as gold standard to train the basecaller. They demonstrate substantial improvement in accurately calling the telomere motifs in nanopore reads using the trained basecaller.
This is an important advance in understanding the current problems that using nanopore reads may cause in analyzing and distinguishing some types of repeat motifs, and also highlights some current limitations of nanopore basecallers. As such, there will be considerable interest in the nanopore sequencing community given the critical role of telomeres in biology and human disease and the potential advances in sequencing and assembly that nanopore methods promise for complete telomere to telomere genomes, it should be of interest to a much wider readership as well.

We are happy to note that the reviewer thinks our work will be of considerable interest to the nanopore sequencing community, and also to a much wider readership.

The data and methods presented are fairly solid and support the main conclusions, but there are several questions/issues that should be addressed so the reader can understand the limits of the current telomere correction methods described.

We are glad to hear that the reviewer thinks our data and methods presented are fairly solid and support the main conclusions. As the reviewer suggested, we have looked in greater depth into the questions/issues that the reviewer raised to help the reader understand the limits of the current telomere correction methods described.

1. The authors identify telomere-containing nanopore reads for further analysis by mapping nanopore reads in the CHM13 dataset back to telomere/subtelomere regions of the CHM13 T2T draft assembly, which included both a HiFi assembly and a nanopore dataset. We need more detail as to how well this nanopore read-mapping actually worked, because traditionally this step has been problematic due to telomere-adjacent segmental duplications that can extend for tens to hundreds of kb from the terminal repeat tract, and single nanopore reads are reported to be somewhat error-prone throughout their read lengths (maybe 93-95 % ??). Was there a size selection to make sure only ultralong reads were used that spanned the segmental duplications? Were there steps taken to identify and screen out reads that might map to multiple telomeres? What was the range of nanopore read coverage for individual mapped telomeres?

The reviewer raised a great point about how read mapping can be affected by telomere-adjacent segmental duplications. Indeed, as the reviewer pointed out, the mapping of reads to the different chromosomal arms has traditionally been problematic due to telomere-adjacent segmental duplications that extend for tens to hundreds of kb from the terminal repeat. Specifically, in the sub-telomeric maps generated by Stong *et al*, Genome Research, 2014 [1], many chromosomal arms have been shown to contain a high degree of paralogy and sequence similarity (>90%) with each other. As such, given the high degree of sequence similarity, read mis-mapping between different chromosomal arms can potentially occur as the reviewer has suggested. That said, we do not think that mis-mapping of reads between different chromosomal arms is a significant issue in the CHM13 sample for the following reasons:-

We first simulated long-reads (mean = 10kb) from the terminal 10kb, 100kb, and 1000kb region of the CHM13 reference genome using PBSIM2 [2] **(Methods)**, and then remapped these reads back to the CHM13 reference sequences using the same mapping parameters as our original analysis. The level of read mis-mapping was then assessed using the paftools.js script distributed as part of minimap2 [3]. Remarkably, under a mapping quality threshold of ≥1, the mapping error rate was only ~0.03-0.3% at each of these terminal regions and for reads ranging in accuracy between 95-99.9% **(Revised Supplementary Figure 5; Reproduced as Response Figure 5)**. Even when a less stringent mapping quality cutoff value of 0 was applied, a relatively low mapping error rate of 0.3-1.2% was observed **(Revised Supplementary Figure 5; Reproduced as Response Figure 5)**. As such, given the relatively low mapping error rate from our read simulation study, our results from read simulation thus indicate that there is minimal level of read mis-mapping between different chromosomal arms in the CHM13 sample.

**Response Figure 5 Mapping error rate of long-reads simulated from terminal ends of the CHM13 reference genome.** Long-reads were simulated from the terminal 10kb, 100kb or 1000kb ends of each chromosome in the CHM13 reference genome. Different error rates for the reads was also assessed, with read accuracy of 95.0%, 98.0%, and 99.9%. Reads were then remapped to the CHM13 reference genome with minimap2 and the corresponding mis-mapping rate of these reads assessed under different mapping quality thresholds.

Next, as the reviewer suggested, we evaluated the sequencing coverage of each chromosomal arms to see if there might be a bias in the number of reads that mapped to each chromosomal arm **(Revised Supplementary Figure 6a; Reproduced as Response Figure 6a)**. We observed a similar number of reads mapped to each arm at the flanking 10kb region, though some arms seem to have slightly inflated number of reads mapped to it, while others seem to have a slightly depleted count. To establish if the variation we observed might be caused by stochastic variation for instance induced by library preparation, we further extend our analysis to the flanking 100kb and 1000kb region where the effect of mis-mapping are likely to be minimized. Interesting, we see that the variation in the fraction of reads mapped to each chromosomal arms in the 10kb, 100kb and 1000kb region was similar to each other **(Revised Supplementary Figure 6b; Reproduced as Response Figure 6b)**, suggesting that the variation that we have observed in the terminal 10kb region can be reasonably explained by stochastic variation induced for instance during library preparation. Together our results indicate that there is minimal effect of read mis-mapping between different chromosomal arms in the CHM13 sample.

Third, we like to highlight that the emphasis of our study was not to look at telomeres precisely on the chromosomal arm level, but to report on the overall phenomenon of repeat calling errors at telomeres. Thus, even if these telomeric reads were mis-mapped to the wrong chromosomal arm, it would not affect our overall observation of these repeat calling errors since the errors on the telomeric region of these reads would still be observed even if the reads were mis-mapped to the wrong chromosomal arm. That said, our results above indicate that there is likely minimal level of read mis-mapping (<1.2%) between different chromosomal arms in the CHM13 sample. We think that this is likely due to the existence of a high quality and fully assembled CHM13 reference genome for us to map the CHM13 nanopore reads onto.

Noting the three reasons above, we conclude that mis-mapping of telomeric reads to the wrong chromosomal arm is likely to be low (<1.2%), and thus unlikely to be a significant issue in our study of the CHM13 sample.

The reviewer also asked if steps were taken to identify and screen out reads that might map to multiple telomeres. As highlighted by our analysis above, the mis-mapping of reads between different chromosomal arms is likely to be low (<1.2%) in the CHM13 sample. Noting the low mis-mapping rate, we do not think that screening out reads that might map to multiple telomeres is going to significantly affect our analysis.

The reviewer further asked if a size selection step was performed to make sure that only ultralong reads were used that spanned the segmental duplications. While we agree with the reviewer that ultra-long reads that span well into the unique regions of each arm can be more accurately mapped to the correct chromosomal arm, our simulation study of long-reads with a mean read length of 10kb can already be mapped to the correct chromosomal arm >98.8% of the time. As such, we do not think it is essential to specifically select for ultralong reads that spans into the unique region of each chromosomal arm for our analysis.

Overall, given the results and reasons presented above, we do not think that read mis-mapping between different chromosomal arms is a significant issue in our study of the CHM13 sample.

**Response Figure 6 Negligible bias in read coverage of each of the chromosomal arms was observed in the nanopore sequencing dataset for the CHM13 sample. (A)** Barplot depicting the number of reads mapped to the flanking 10kb region of each chromosomal arm of the CHM13 reference genome. **(B)** Boxplot depicting the fraction of reads that mapped to each chromosomal arm. The fraction of reads mapped to each arm was calculated by dividing the number of reads observed at each of the arm by the total number of reads across all chromosomal arms for the corresponding terminal region size of 10kb, 100kb, or 1000kb. Similar levels of variation was observed for reads mapped to the terminal 10kb region where most mapping errors are expected, as the terminal 1000kb region where mapping errors are less likely to occur.

2. The centromeric end of the human terminal repeat tract typically contains telomere-like sequences similar but non-identical to the canonical repeats (e.g., (TCAGGG)n, TTGGGG, etc); these are usually telomere-specific and often allele-specific. Does the basecaller perform well enough to detect these amidst the canonical motifs?

Here, the reviewer raised an important and interesting point about the existence of telomere-like sequences (e.g. (TCAGGG)_n_, (TTGGGG)_n_ sequences) on the centromeric end of the human terminal repeat tract, and asked if the basecaller performs well enough to detect these sequences admidst the canonical motifs. Indeed, the existence of telomere-like sequences had been reported previously [4–6]. In our understanding, there are three main types of telomere-like repeat sequences, namely the c-type repeats (TCAGGG)_n_, g-type repeats (TGAGGG)_n,_ and_,_ j-type repeats (TTGGGG)_n_ [7]. Other potential telomere-like sequences were also reported in a recent study by Grigorev et al [8]. However, as (TCAGGG)_n_, g-type repeats (TGAGGG)_n,_ and_,_ j-type repeats (TTGGGG)_n_ repeats are more commonly assessed, we will focus on analysis on these three types of repeats.

Here, we address the reviewer’s queries by two approaches: First, we identified regions in the CHM13 reference genome with telomere-like sequences and established if these regions were basecalled correctly in Nanopore data generated from the CHM13 cell line. Second, we revisited results of the pair-wise simulation experiments of current profiles presented in this study to assess if these telomere-like sequences might share similar current profiles as other repeat sequences.

First, to establish if the telomere-like regions were basecalled correctly, we first analyzed the CHM13 reference genome to identify where these regions might be. We were able to identify 7 regions with (TCAGGG)_n_, 7 regions with (TGAGGG)_n_, and 5 regions with (TTGGGG)_n_ repeats that are at least 100 bp in length. Notably all these regions exist within 10kb of the terminal end of each chromosomal arm. We then visualized each of these regions in IGV, and were able to identify repeat calling errors at these telomere-like repeat sequences to different degrees at each of these types of repeats **(Revised Supplementary Figure 14; Reproduced as Response Figure 7)**.

What is particularly significant is that we also noticed that these telomere-like repeat sequences could also be miscalled into repeat sequences of other repeat lengths. For instance, we observed that the 6-mer (CCCTCA)_n_ repeats could get miscalled into the 5-mer (CCTCA)_n_ repeat sequence **(Revised Supplementary Figure 14a; Reproduced as Response Figure 7a)**. (CCCTGA)_n_ and (TCAGGG)_n_ 6-mer repeats could also get miscalled into (CCTGA)_n_ 5-mer repeats and (TCAGGGG)_n_ 7-mer repeats respectively **(Revised Supplementary Figure 14b; Reproduced as Response Figure 7b)**. Further, the 6-mer (TTGGGG)_n_ repeat was observed to be miscalled into the 7-mer (TTGGGGG)_n_ repeats **(Revised Supplementary Figure 14c; Reproduced as Response Figure 7c)**. Together, these suggests that the repeat miscalling errors are not restricted to 6-mers (which is the approximate number of nucleotides detected by the nanopore at each time) and can be slightly shorter or longer.

We then explored current profiles for these repeats (10 consecutive repeats) using known current values for each 6-mer repeats **(Revised Supplementary Figure 15; Reproduced as Response Figure 8)**. Remarkably, even though these repeat sequences were of different length, we see that these sequences can still share a highly similar current profile **(Revised Supplementary Figure 15a,b,e,g; Reproduced as Response Figure 8a,b,e,g).** Together, these suggests that repeat sequences of different lengths can share similar current profiles and be miscalled between each other.

Second, we revisited results of the pair-wise simulation experiments presented in our original manuscript. We then assessed each of these three types of repeats to see if they might share similar current profiles to other types of 6-mer repeats. Indeed, using a Pearson correlation cutoff of ≥0.99, we see that (TCAGGG)n repeats share a similar current profile as 7 other types of repeats **(Revised Supplementary Figure 16a; Reproduced as Response Figure 9a)**, (TGAGGG)_n_ share a similar current profile as 3 other types of repeats **(Revised Supplementary Figure 16b; Reproduced as Response Figure 9b)**, and that (TTGGGG)n share a similar current profile as 4 other types of repeats **(Revised Supplementary Figure 16c; Reproduced as Response Figure 9c)**.

Together, our results suggest that the Nanopore basecaller (Bonito) does miscall these telomere-like sequences to some degree in the CHM13 dataset, and can also miscall these regions into sequences of different lengths.

**Response Figure 7 IGV screenshots depicting repeat calling errors observed on telomere-like repeat sequences in the CHM13 dataset. (a)** Repeat calling error observed at the (TGAGGG)_n_ g-type repeat region in the CHM13 sample. The region represented corresponds to chr1:248,385,364-248,385,449. **(b)** Repeat calling error observed at the (TCAGGG)_n_ c-type repeat region in the CHM13 sample. The region represented corresponds to chr1:2,381-2,420. **(C)** Repeat calling error observed at the (TCGGGG)_n_ j-type repeat region in the CHM13 sample. The region represented corresponds to chr1:2,895-2,941.

**Response Figure 8 Simulated current profiles for 10 consecutive repeats of the telomere-like repeat sequences, and observed repeat calling errors. (a-c)** Simulated current profiles for repeat calling error repeats observed at (TGAGGG)_n_ telomere-like repeat sequences. **(d-f)** Simulated current profiles for repeat calling error repeats observed at (TCAGGG)_n_ telomere-like repeat sequences. **(g)** Simulated current profiles for repeat calling error repeat observed at (TTGGGG)_n_ telomere-like repeat sequences. The three types of telomere-like repeat sequences are colored in blue, while the repeat calling errors are labelled in red. The length of repeat sequences presented in each of the panel are also highlighted in the legend. Note that slight horizontal scaling (<5%) was applied to the red current profiles for panels b and e to allow the current profiles to align better with the blue current profiles. The scaling factor can be read off the from the last red dot of each of these plots.

**Response Figure 9 Repeats with predicted similarity in current profiles to the three types of telomere-like repeat sequences.** Repeats that have predicted similarities in current profiles to **(a)** (TCAGGG)_n_ **(b)** (TGAGGG)_n_ and **(c)** (TTGGGG)_n_ are indicated in each of the plots.

**References**

1. Stong N, Deng Z, Gupta R, Hu S, Paul S, Weiner AK, et al. Subtelomeric CTCF and cohesin binding site organization using improved subtelomere assemblies and a novel annotation pipeline. Genome Res [Internet]. 2014;24:1039–50. Available from: http://genome.cshlp.org/lookup/doi/10.1101/gr.166983.113

2. Ono Y, Asai K, Hamada M. PBSIM2: a simulator for long-read sequencers with a novel generative model of quality scores. Peter R, editor. Bioinformatics [Internet]. 2021;37:589–95. Available from: https://academic.oup.com/bioinformatics/article/37/5/589/5911629

3. Li H. Minimap2: pairwise alignment for nucleotide sequences. Birol I, editor. Bioinformatics [Internet]. 2018;34:3094–100. Available from: https://academic.oup.com/bioinformatics/article/34/18/3094/4994778

4. Coleman J, Baird DM, Royle NJ. The Plasticity of Human Telomeres Demonstrated by a Hypervariable Telomere Repeat Array That Is Located on Some Copies of 16p and 16q. Hum Mol Genet [Internet]. 1999;8:1637–46. Available from: https://academic.oup.com/hmg/article-lookup/doi/10.1093/hmg/8.9.1637

5. Lee M, Hills M, Conomos D, Stutz MD, Dagg RA, Lau LMS, et al. Telomere extension by telomerase and ALT generates variant repeats by mechanistically distinct processes. Nucleic Acids Res [Internet]. 2014;42:1733–46. Available from: https://academic.oup.com/nar/article-lookup/doi/10.1093/nar/gkt1117

6. Varley H, Pickett HA, Foxon JL, Reddel RR, Royle NJ. Molecular characterization of inter-telomere and intra-telomere mutations in human ALT cells. Nat Genet [Internet]. 2002;30:301–5. Available from: http://www.nature.com/articles/ng834z

7. Feuerbach L, Sieverling L, Deeg KI, Ginsbach P, Hutter B, Buchhalter I, et al. TelomereHunter – in silico estimation of telomere content and composition from cancer genomes. BMC Bioinformatics [Internet]. 2019;20:272. Available from: https://bmcbioinformatics.biomedcentral.com/articles/10.1186/s12859-019-2851-0

8. Grigorev K, Foox J, Bezdan D, Butler D, Luxton JJ, Reed J, et al. Haplotype diversity and sequence heterogeneity of human telomeres. Genome Res [Internet]. 2021;31:1269–79. Available from: http://genome.cshlp.org/lookup/doi/10.1101/gr.274639.120

**Second round of review**

**Reviewer 1**

The authors have conducted extensive analysis and addressed my concerns. I am very happy to see the revised manuscript. I think the work would be of great interest to researchers working on long-reads and to other genome biology readers as well.

**Reviewer 2**

Tan et al. carry out significant new analyses to address the reviewer comments in this revised manuscript, further supporting their key observations of strand-specific nanopore sequencing basecalling errors in telomere sequences that vary in type and severity upon the basecaller used and the specific nanopore sequencing platform and conditions. The errors are caused by the high similarity of current profiles between true telomere sequences and their cognate artifactually called repeat sequences. The authors show further that these types of errors also occur in the telomere-like repeats adjacent to (TTAGGG)n tracts, as well as in many non-(TTAGGG)n telomeres sequences in a wide range of organisms. The work provides a rich analysis of nanopore-derived telomere sequences and suggests these types of basecalling errors are likely to occur in nanopore reads from other similarly organized tandem repeats elsewhere in this genome. In short, the revisions have provided additional data and detail, and significantly improved an already interesting and important paper.
